# Improved Imaging Surface for Quantitative Single-Molecule Microscopy

**DOI:** 10.1021/acsami.4c06512

**Published:** 2024-07-09

**Authors:** Yu P. Zhang, Evgeniia Lobanova, Asher Dworkin, Martin Furlepa, Woo Suk Yang, Melanie Burke, Jonathan X. Meng, Natalie Potter, Renata Lang Sala, Lakmini Kahanawita, Florence Layburn, Oren A. Scherman, Caroline H. Williams-Gray, David Klenerman

**Affiliations:** † Department of Chemistry, University of Cambridge, Lensfield Road, Cambridge CB2 1EW, U.K.; ‡ UK Dementia Research Institute at Cambridge, Cambridge CB2 0XY, U.K.; § Department of Clinical Neurosciences, University of Cambridge, Cambridge CB2 0PY, U.K.

**Keywords:** imaging surface, surface passivation, surface chemistry, single-molecule microscopy, protein aggregates, super-resolution microscopy

## Abstract

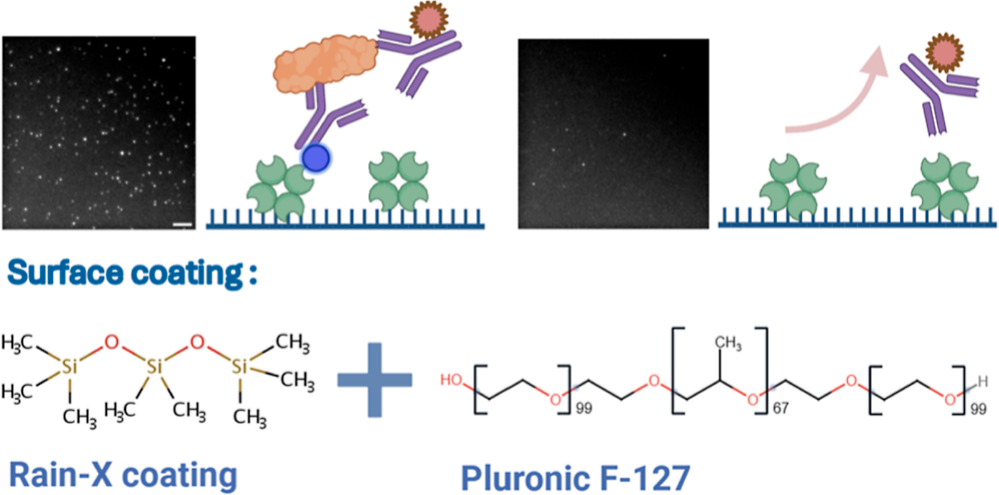

Preventing nonspecific binding is essential for sensitive surface-based quantitative single-molecule microscopy. Here we report a much-simplified RainX-F127 (RF-127) surface with improved passivation. This surface achieves up to 100-fold less nonspecific binding from protein aggregates compared to commonly used polyethylene glycol (PEG) surfaces. The method is compatible with common single-molecule techniques including single-molecule pull-down (SiMPull), super-resolution imaging, antibody-binding screening and single exosome visualization. This method is also able to specifically detect alpha-synuclein (α-syn) and tau aggregates from a wide range of biofluids including human serum, brain extracts, cerebrospinal fluid (CSF) and saliva. The simplicity of this method further allows the functionalization of microplates for robot-assisted high-throughput single-molecule experiments. Overall, this simple but improved surface offers a versatile platform for quantitative single-molecule microscopy without the need for specialized equipment or personnel.

## Introduction

**Figure 1:**
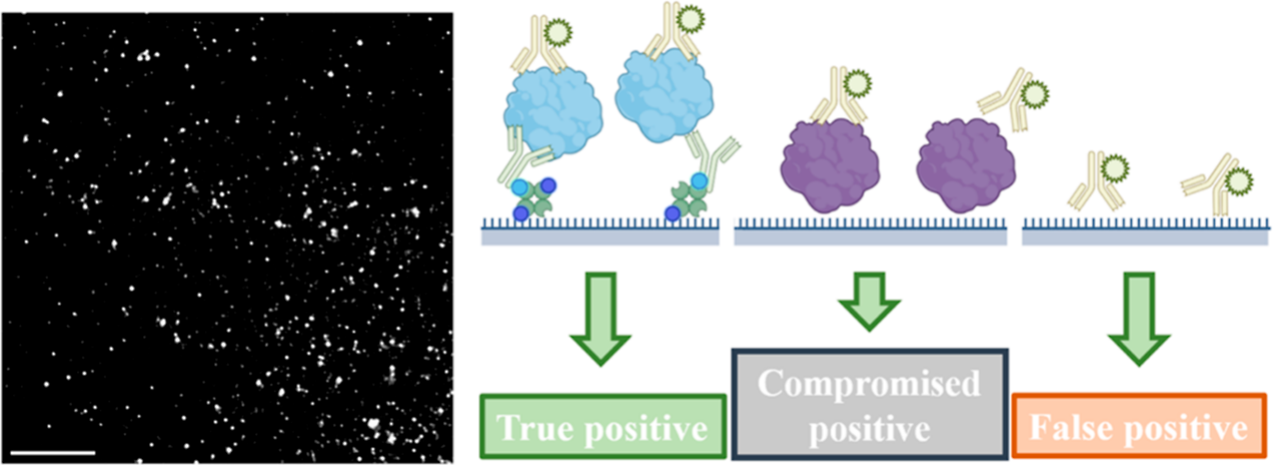
True and false positive signals in surface-based SMFM. In surface-based SMFM, both true and false positive signals can be observed. True positive signals are generated when there is specific binding between the surface capture and detection probes, maximizing the assay’s specificity. However, if the surface fails to capture the target specifically, the detection specificity will be compromised. Direct nonspecific binding of the detection probe to the surface can result in false positive signals, which can significantly bias the measurements. A representative SMFM image illustrates SiMPull imaging of α-syn aggregates derived from human serum. AF647 labeled Syn211 antibody was used for visualization. Scale bar: 10 μm.

Surface-based single-molecule fluorescence microscopy (SMFM) is a widely utilized quantitative method in various biological studies. It is commonly employed for sensitive protein characterization, DNA quadruplex visualization, antibody affinity screening, DNA-based nanostructure mapping and many other applications.^1−8^ The surface-immobilization of single molecules enables precise mapping of their intensity and localizations, making the method an ideal tool to characterize morphologically heterogeneous species such as protein aggregates.^3,6^ However, SMFM is sensitive to fluorescent backgrounds, which usually result from nonspecific binding of the fluorescent molecules. The fluorescent molecules used for imaging may bind to the surface itself, instead of the intended target on the surface, as depicted in Figure 1. These backgrounds compromise the imaging quality and sample quantification. Therefore, it is essential to have imaging surfaces and fluorescent probes that generate low background signals. Systems like single aggregate visualization enhancement^9^ and aptamer DNA-based point accumulation for imaging in nanoscale topography (AD-PAINT),^6,10,11^ have been devised to facilitate low-background single-molecule imaging. However, these systems use plain glass surfaces for imaging and their detection probes need to have minimal binding with the glass surface. This limitation leads to a narrow selection of compatible probes and restricts their further application.

A more common strategy to minimize nonspecific binding is selectively passivating the imaging surfaces. This is typically achieved through surface modifications, which involve a passivation coating and specific binding site deposition. Other active strategies, like the acoustic wave and shearing forces-based cleaning methods,^12^ are usually aggressive and not compatible with sensitive SMFM. The passivation coating is designed to impede nonspecific binding, while the specific binding sites facilitate selective immobilization of the targets. This combination ensures a high degree of detection specificity and reduces background interference, thus improving the quality of the imaging and the accuracy of sample quantification. PEG coating is one of the most common methods for surface passivation, due to its high biological compatibility and resistance to nonspecific bindings.^13,14^ The PEG molecules can covalently bind to the surface and form passivation layers. Common coating strategies include conjugating PEG-silane molecules onto hydroxyl-activated surfaces or attaching PEG-*N*-Hydroxysuccinimide molecules to amine-functionalized surfaces.^3,5,10,13−15^ Biotin-conjugated PEG molecules can be used to coat the coverslips following the same approach offering specific binding sites. However, PEG surfaces do not perform well with concentrated samples,^5^ as highly concentrated molecules can bind to the surface regardless of the surface capture agents, compromising the capture specificity of the surface and limiting its applications. Moreover, these methods require extensive processing with hazardous chemical reagents, such as piranha solution and (3-Aminopropyl) triethoxysilane.^3,10,13,14^ Hence, these surface coatings are often less available in biology or physics-oriented laboratories due to safety concerns and limitation of expertise. While a few recent studies have introduced self-assembly layers to improve surface passivation instead of using covalent PEG methods,^5,16^ these approaches still require extensive surface processing with hazardous chemicals, including concentrated hydrochloric acid, Sigmacote or dichlorodimethylsilane.^5,16^ The improved passivation performance with these methods is more achievable in chemistry laboratories and remains less optimal for biology or physics-oriented laboratories.

In this study, we present a novel surface passivation method that demonstrates enhanced antifouling properties with a much-simplified manufacturing process. The surface passivation in this study is achieved through the self-assembly of amphipathic Pluronic F-127 polymers and the deposition of NeutrAvidin onto a hydrophobic coating.^16,17^ This approach effectively modifies the surface properties, rendering it resistant to biofouling and ensuring optimal performance. We used Rain-X, a relatively safe household chemical, to process the surface instead of more hazardous chemicals. Rain-X contains a collection of Polydimethylsiloxane (PDMS) fragments and produces a hydrophobic base coating for further deposition of Pluronic F-127 and NeutrAvidin.^16−18^ Our method has several major improvements compared with established protocols: (1) the surface has up to 100-fold less nonspecific binding from protein aggregates compared with the PEG surface. (2) The surface fabrication requires much less time to complete and does not need special surface activation. (3) The coating processing is environmentally friendly, and no chemical waste is produced. Additionally, all the materials used in this work are readily available for purchase, eliminating the necessity to synthesize or modify any specialized molecules, unlike several previous reports.^16,19^ We demonstrate that this surface is compatible with various common techniques in SMFM, such as SiMPull, direct stochastic optical reconstruction microscopy (dSTORM), DNA-based point accumulation for imaging in nanoscale topography (DNA-PAINT), visualization of single exosomes and antibody affinity screening.^1,2,20−23^ This improved method not only provides an alternative to PEG surfaces but also serves as a superior tool for characterizing protein aggregates, as PEG surfaces tend to exhibit high nonspecific binding with highly heterogeneous species. Moreover, the simplicity of the method further enables the functionalization of microplates for robot-assisted large-scale single-molecule sensing. In summary, the RF-127 surface exhibits significantly improved passivation and simplified manufacturing compared to the PEG surface, making it a promising and versatile platform for various single-molecule assays.

## Results and Discussion

### Compare RF127 Surface with PEG Surface

To evaluate the anti-biofouling characteristics of the surface, we measured the nonspecific adsorption of various biomolecules on both the RF-127 and PEG surfaces. We included IgG antibodies, DNA aptamers and several recombinant protein aggregates in the tests to comprehensively assess the quality of surface passivation. IgG antibodies and DNA aptamers are commonly used detection probes in biological experiments, while protein aggregates are highly correlated to major neurodegenerative diseases.^3,6,24−28^ Compared to monomeric proteins, the structure of aggregated proteins is notably more intricate and heterogeneous, leading to more significant nonspecific interactions with the imaging surface. As a result, they require stringent surface passivation to mitigate nonspecific signals. We first evaluated the fluorescent background of the surface (assessed using 488, 561, and 638 nm wavelength lasers). The properly prepared surface showed a low background and did not interfere with the quantification of specific signals. (see Figure S1 in Supporting Information). The passivation of surfaces was quantified by counting the number of nonspecifically adsorbed molecules on the passivated surface. Identical contrast was applied to representative image pairs to illustrate the number of detected single molecules, however, paired images with divergent brightness may suffer from visualization defects (See Figure S2 in Supporting Information). We have provided adjusted versions of all paired representative images to ensure the visibility of all single molecules (See Figure S2 in Supporting Information).

**Figure 2:**
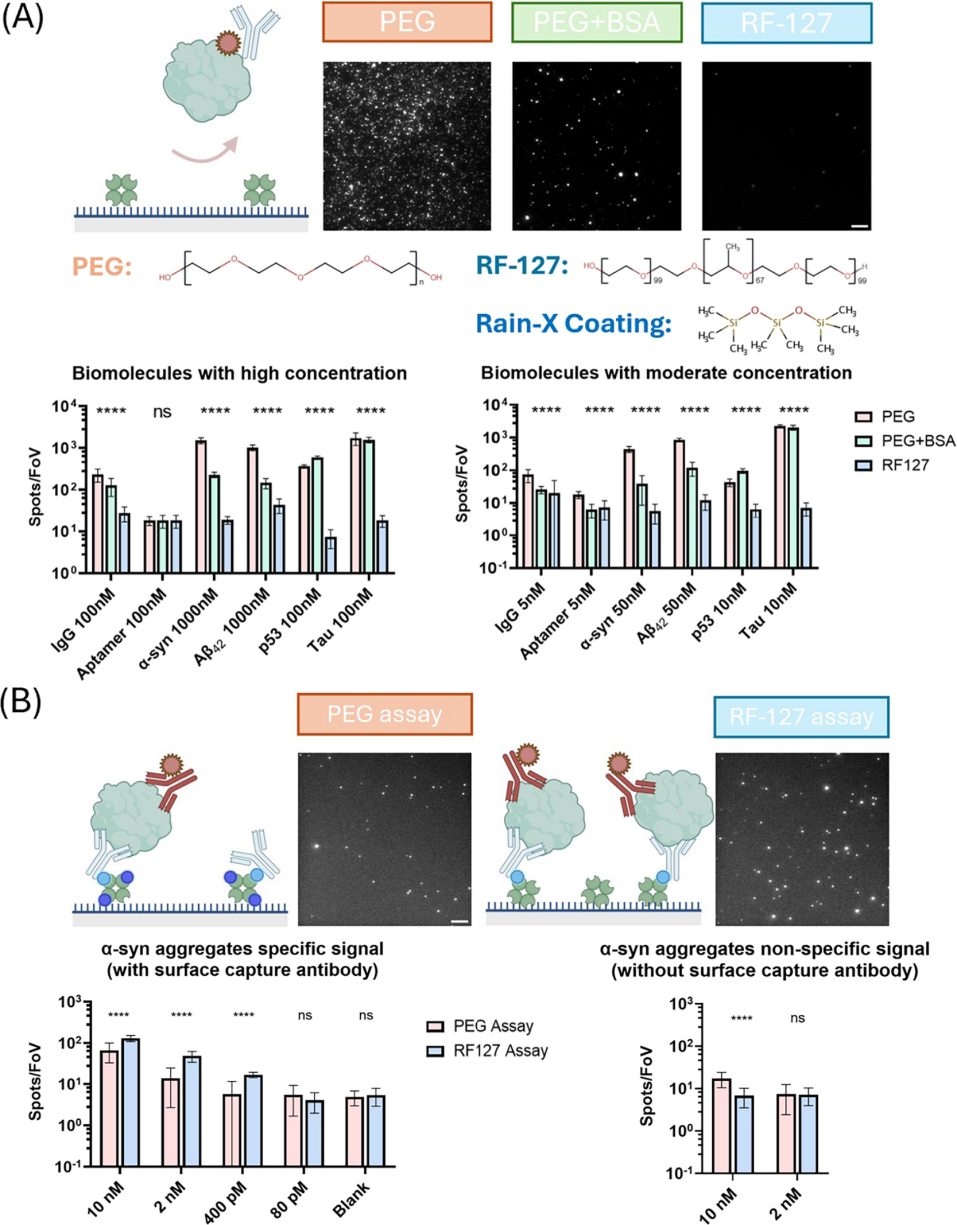
Comparison of surface passivation and specific affinity of the RF-127 surface with a PEG surface. (A) Passivation comparison. Briefly, the PEG surface uses covalently bonded PEG for passivation and biotin-conjugated PEG for specific binding. The RF-127 surface employs a combination of Pluronic F-127 self-assembly and NeutrAvidin deposition on Rain-X hydrophobic coating, for both passivation and specific binding. The images present the nonspecific binding of 1000 nM α-syn aggregates on PEG. PEG-BSA and RF-127 surface. The aggregates were visualized with the AF-647 labeled syn-211 antibody. Identical contrast was applied to the images. Scale bar: 5 μm. Surface passivation was quantified by the number of nonspecific bound IgG antibodies, DNA aptamer, α-syn, Aβ, tau and p53 aggregates onto the surfaces. IgG antibodies and DNA aptamers were covalently labeled with AF647. Protein aggregates were labeled by AF647 conjugated antibodies (See Table S1 for details of antibodies). Note that the *y*-axis is presented on a Log10 scale. Error bars: s.d. (*n* = 16, from 16 fields of views). **** demotes *P* < 0.0001, one-way ANOVA. Each field of view (FoV) contains an area of ∼2500 μm^2^. The concentration of aggregates is in monomer-equivalents and these aggregates are recombinant species. (B) Specific affinity comparison between the PEG and RF-127 surface. Specific antibodies were immobilized onto each surface to capture recombinant α-syn aggregates. The PEG surface was blocked with BSA. The aggregates were captured and visualized with syn-211 antibody (AF-647). Error bars: s.d. (*n* = 16, from 16 fields of views). **** demotes *P* < 0.0001, unpaired *t*-test. Each FoV contains an area of ∼2500 μm^2^. The concentration of aggregates is in monomer-equivalents and these aggregates are recombinant species.

As shown in Figure 2, the RF-127 surface has superior performance to the PEG surface in most tests. The synergistic use of NeutrAvidin and RF-127 optimizes surface passivation while ensuring an abundance of specific binding sites. In the tests using concentrated samples (lower left panel of Figure 2A), RF-127 has approximately 100-fold less nonspecific binding from sticky protein aggregates of tau and p53. These protein aggregates generated the highest level of nonspecific binding. The presence of bovine serum albumin (BSA) blocking agents on the PEG surfaces does not alter this result. In the case of less sticky molecules, such as α-syn aggregates, amyloid beta (Aβ) aggregates and IgG antibodies, the RF-127 surface also demonstrates much lower nonspecific binding compared to the PEG surface without using BSA for blocking. Specifically, the RF-127 surface exhibits approximately 80-fold less nonspecific binding for α-syn aggregates, 50-fold less for Aβ aggregates and 10-fold less for IgG antibodies when compared to the unblocked PEG surface. Though BSA blocking can enhance the passivation of PEG surfaces, the RF-127 surface still has a 5-fold less nonspecific binding for α-syn aggregates, 3-fold less for Aβ aggregates and 3-fold less for IgG antibodies in comparison to the blocked PEG surface. When using a lower concentration of biomolecules, the RF-127 surface maintains significantly reduced nonspecific binding as shown in the lower right panel of Figure 2B, indicating its superior performance in preventing unwanted interactions compared to the PEG surface. Interestingly, a decrease in the concentration of recombinant tau aggregates did not reduce the level of nonspecific adsorption. This observation suggests recombinant tau aggregates are exceptionally prone to bind surface nonspecifically even at lower concentrations. The PEG surface is not optimal for the characterization of these molecules as the high-level nonspecific interactions can compromise the detection specificity, resulting in poor image quality. (see Figure S3 in Supporting Information). This highlights the need for effective passivation strategies, such as the RF-127 surface, to minimize nonspecific interactions and ensure accurate characterization of these sticky protein aggregates. We further conducted additional tests to evaluate the passivation effectiveness of a recently reported method^16^ that employs a similar self-assembly strategy (Sigmacote + F127) with adhesive tau aggregates. Both methods exhibit comparable antifouling performance, yet RF-127 stands out for its notably simpler implementation. (See Figure S4 in Supporting Information).

Additionally, we conducted further testing to assess the specific affinity of the RF-127 surface. Results indicate that RF-127 was capable of capturing more α-syn than the PEG surface when the diluted sample was applied. Under the tested conditions, the RF-127 surface was able to immobilize five times more antibodies onto the surface compared to the PEG surface (See Figure S5 in Supporting Information). The RF-127 surface has a significantly higher number of captured aggregates until the sample concentration drops below 400 pM. The antibody density on the RF127 surface was measured to be around 720/μm^2^ (equivalent to 1,800,000 per FoV), whereas the PEG surface had a density of 150/μm^2^ (equivalent to 360,000 per FoV). The higher antibody surface density of RF-127 can contribute to improved detection sensitivity, particularly considering that protein aggregates are often present at low concentrations in biofluids.^6,8^ Moreover, when no capture antibodies were present, both surfaces demonstrated good resistance to nonspecific sample binding. The RF-127 surface exhibited significantly better performance when a higher concentration of the sample (10 nM) was applied. However, when the sample concentration was diluted, both PEG and RF-127 surfaces exhibited similar good performance.

**Figure 3:**
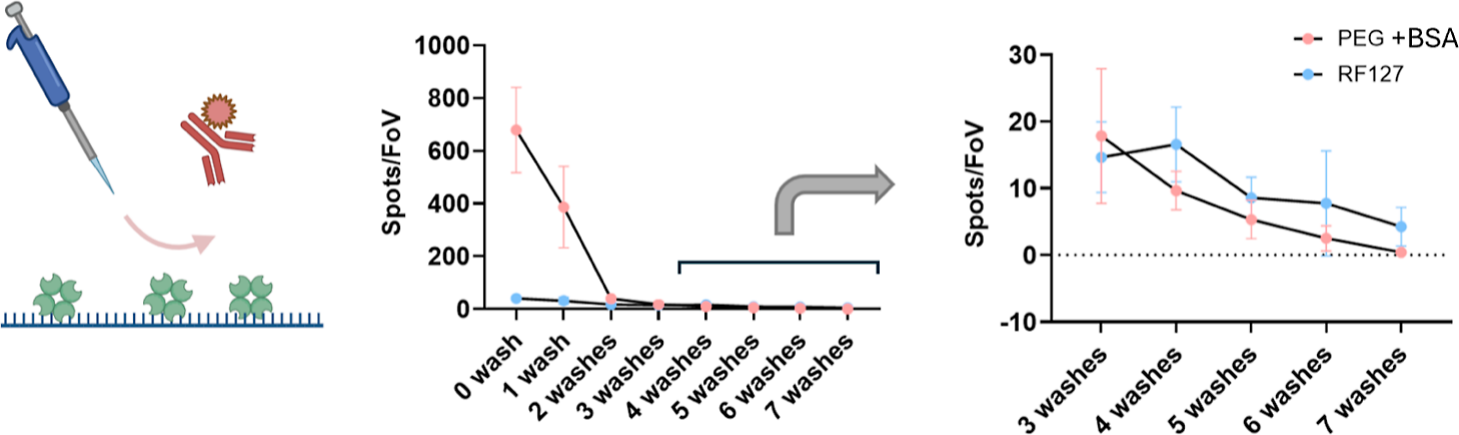
Effect of different washing steps on reducing nonspecific signals on the PEG and RF-127 surfaces. One washing step involved pipetting 10 μL of PBST into the sample well and then aspirating it back out. The nonspecific signals were created by incubating the surfaces with 10 nM AF647-IgG for 15 min. Similar to the results shown in Figure 2, the PEG surface (with BSA blocking) and RF-127 surface have similar passivation performance against IgG when a lower concentration was applied. PEG surfaces typically require multiple washing steps to effectively reduce nonspecific binding and minimize background signals. On the other hand, RF-127 surfaces demonstrate a low level of nonspecific binding without the need for extensive washing. Vigorous washing (>4 washes) can remove almost all nonspecific bound IgG antibodies on the PEG surface, while the RF-127 surface retained slightly higher signals. However, most single-molecule immunofluorescence assays only include 2–3 washes, since vigorous washes can potentially remove positive signals.^1,3,10,20^ Error bars: s.d. (*n* = 12). Each FoV contains an area of 2500 μm^2^.

The antifouling properties of RF-127 and PEG surfaces have different physical origins. Like PEG, Pluronic F-127 is a widely used reagent for creating antifouling interfaces. This amphiphilic molecule has both hydrophilic and hydrophobic regions in its structure. When interacting with hydrophobic surfaces, the hydrophobic core of Pluronic F-127 facilitates the formation of a self-assembly layer while the poly(ethylene oxide) (PEO) tails of Pluronic F-127 extend outward. These PEO tails create a hydrophilic environment that repels nonspecific biomolecular interactions, preventing their adsorption onto the surface. We observed a reduction in water contact angle upon the Pluronic F-127 coating on the Rain-X surface, suggesting the successful formation of the F-127 layer. (See Figure S6 in Supporting Information). Pluronic F-127 is bigger (MW ∼ 12.6 kDa) than most PEG molecules reported in surface passivation studies (MW ∼ 5 kDa), featuring two PEG chains instead of one.^1,3,14^ This creates a higher local density of PEG chains even if the average molecular density is the same. F-127 can also form a brush-like conformation enhancing its ability to effectively resist nonspecific binding.^29^ Additionally, the covalent surface attachment of PEG molecules commonly depends on active esters, known for their short half-life, potentially resulting in a decreased deposition density.^30^ Therefore, nonspecific binding to the Pluronic F-127 layer would be more unfavorable, resulting in low nonspecific binding. In comparison to the PEG surface, RF-127 requires fewer washing steps to remove nonspecific signals to reduce the noise to a similar level, as shown in Figure 3. Only vigorous washing can remove the majority of the nonspecific bindings on the PEG surface, while RF-127 does not require such a process. For specific binding, the effect of vigorous washings depended on the probe used. High affinity probes showed no reduction while lower affinity probes showed a reduction in signal, after vigorous washing. (See Figure S7).

### Single-Molecule Applications of RF-127 Surface

**Figure 4:**
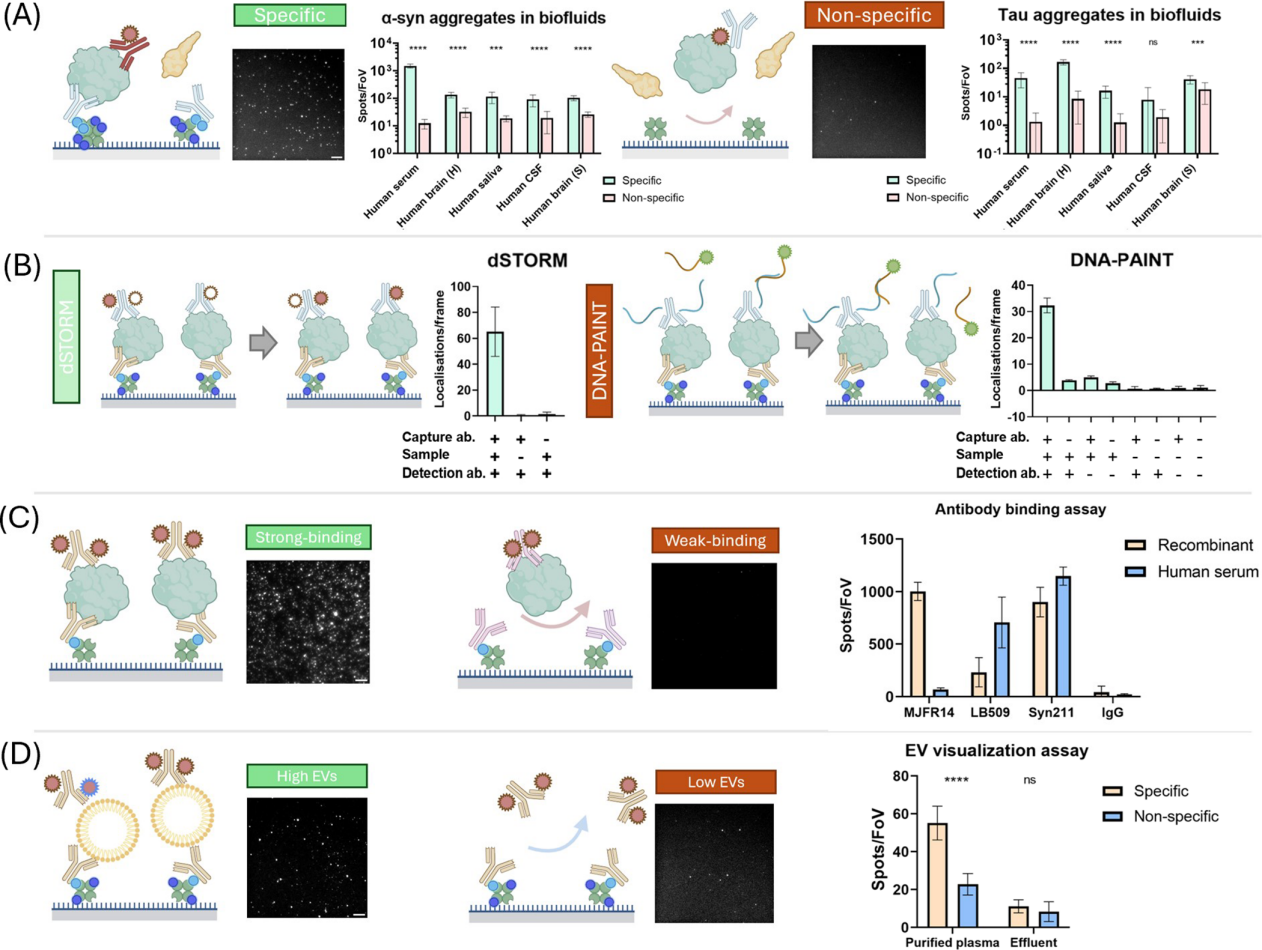
Applications of the RF-127 surface (A). RF-127 based SiMPull. Briefly, protein aggregates are captured onto the surface via the immobilized antibodies, and then visualized with AF647-labeled detection antibodies. When the capture antibody is absent, the surface will only adsorb a low level of aggregates via nonspecific interaction. Human saliva SiMPull images were used as representative data. Scare bar: 5 μm. Error bars: s.d. (*n* = 9–16). Brain samples (H) and (S) denote samples from different extraction methods. See Supporting Information for details. **** demotes *P* < 0.0001, *** demotes 0.0001 < *P* < 0.001, unpaired *t*-test. (B) Schematic of dSTORM (left), DNA-PAINT (right) imaging with the RF-127 surface, and the number of localizations under different conditions. Briefly, both methods use single-molecule localization microscopy to temporally separate unresolvable adjacent fluorophores under the diffraction limit. dSTORM utilizes the photoswitching of dyes while DNA-PAINT relies on the transient binding of dye-labeled imager. Localizations were collected via dSTORM and DNA-PAINT under different conditions. Only the all-positive condition was able to give a high number of single-molecule localizations. Error bars: s.d. (*n* = 3, each replicate consists of 3000 frames). A super-resolved α-syn aggregate (dSTORM, AF647-labeled Syn211 antibody as probe) was used as the representative image. No statistical tests were performed as all control conditions have much less (<10 fold) signal than positive. (C) Schematic of the RF-127-based antibody binding screening assay and the number of detected aggregates on RF-127 surfaces with different antibody pairs. Briefly, since different antibodies possess varying affinity against aggregates, the number of aggregates captured onto the surface can be used to quantify the antibody affinity. LB509 antibody (left) and MJFR14 (right) antibody binding in serum were used as representative images. Scale bar: 10 μm. Error bars: s.d. (*n* = 16). **** demotes *P* < 0.0001, unpaired *t*-test. (D) Visualization of single exosomes purified from plasma using RF-127 surface. Briefly, human plasma was separated into a high exosome level portion (purified plasma) and a low exosome level portion (effluent) using a commercial exosome isolation kit. We only detected high signals from purified plasma with both capture and detection antibodies. The exosomes are captured via anti-CD-63 antibody and visualized by AF647-anti-CD-81 antibody. Scare bar: 5 μm. Each FoV contains an area of 2500 μm^2^. Error bars: s.d. (*n* = 9). **** demotes *P* < 0.0001, unpaired *t*-test.

We first performed SiMPull, a commonly used single-molecule quantitative assay for biological measurements, to demonstrate the application of RF-127 surface in SMFM.^3,10,20,31,32^ The SiMPull assay is capable of immobilizing the biomolecule of interest in a coverslip at coverages so that individual biomolecules are distinguishable for subsequent microscopic imaging. This method has been used to characterize protein aggregates present in human serum, saliva, and brain extracts, demonstrating its potential as both a diagnostic platform and a tool for exploring disease molecular mechanisms. Additionally, the brightness of individual protein aggregates correlates with their size.^3,8,33^Figure 4A illustrates the fundamental working principles of SiMPull, encompassing two key aspects: (1) specific capture and visualization of the target of interest in the imaging surface, and (2) elimination of nonspecific signals generated on the surface. The detection of specific targets is achieved through a combination of capture and detection agents, with antibodies commonly employed as SiMPull’s capture and detection agents. Simultaneously, surface passivation serves to reject unwanted signals. This characteristic makes SiMPull an ideal method to characterize species with heterogeneous single-molecule features, such as protein aggregates.^8^ We measured α-syn and tau aggregates from a wide range of human samples, including human saliva, CSF, brain extracts and serum, to assess the compatibility of RF-127-based SiMPull with complex samples. As shown in Figure 4A, the RF-127 surface is able to capture α-syn and tau aggregates specifically from a wide range of samples with capture antibodies. The absence of capture antibodies significantly reduces the signal level, suggesting the surface has a strong resistance to the nonspecific adsorption of target molecules. In comparison to the full assay (with both capture and detection antibodies), we achieved at least 10-fold less signal in most capture controls (RF-127 surface without capture agents) apart from tau aggregates in the soluble fraction of brain extracts (brain S.). The nonspecifically adsorbed tau aggregates from soaked brain samples on the RF-127 surface are 3-fold less than those captured via specific interactions. The tau aggregates signal from human CSF is similar to the background, demonstrating the need for further optimization. We also assessed the stability of the fluorescence signal when using the RF-127 surface. We observed a comparable signal after 24 h indicating that RF-127 is suitable for long imaging sessions without significant signal decay. (See Figure S8 in Supporting Information). Moreover, since the capture affinity of the surface is controlled via the amount of NeutrAvidin deposited, it is possible to adjust the capture affinity by modifying NeutrAvidin incubation time (See Figure S8 in Supporting Information). NeutrAvidin deposits on PDMS-based hydrophobic surfaces with a strong affinity and offers stable binding sites.^17^ Most PEG surfaces, on the other hand, covalently deposit the biotin molecules and are made well in advance of biological experiments. In contrast, the deposition of NeutrAvidin on the RF-127 surface is carried out on the day of biological experiments, allowing levels to be easily tuned depending on the sample.

Extensive reproducibility tests were performed to ensure the reliability of the RF-127 platform. We measured recombinant α-syn samples in more than 300 individual imaging wells to ensure RF-127 can produce consistent results (See Figure S8 in Supporting Information). During our tests, we observed that coverslips can be effectively cleaned using solvents as an alternative to plasma cleaners, facilitating the deployment of this method in various laboratory settings.

We also tested super-resolution imaging on the RF-127 surface. SMFM-based super-resolution imaging is a widely used technique to characterize samples with a resolution down to 20 nm. These methods have been used in several studies to characterize protein aggregates from various human samples and measure their size distribution.^3,6,10,34^ We applied dSTORM and DNA-PAINT, two widely used super-resolution techniques, to demonstrate their compatibility with the RF-127 surface. Our results in Figure 4B demonstrated that both dSTORM and DNA-PAINT were able to characterize human serum α-syn aggregates captured by the functionalized RF-127 surface. We only observed a large number of localizations when the capture antibody, sample and detection antibody were all presented. (See Figure S10 for representative single molecule localization figures). Although both super-resolution microscopy methods have been widely used, they have different challenges in surface-based imaging. dSTORM requires a special imaging buffer (usually containing an oxygen scavenger system and reducing agents) to maintain photoswitching, while DNA-PAINT needs additional DNA imaging strands to generate blinking. Therefore, the imaging surface needs to have chemical resistance to the imaging buffer (for dSTORM) as well as low biofouling with the DNA imaging strands (for DNA-PAINT). As shown in Figure 4B, both methods are applicable to the RF-127 surface, suggesting that the RF-127 surface has sufficient chemical resistance to the dSTORM buffer and low binding efficiency to the DNA-PAINT imaging strands.

We then tested single-molecule antibody affinity screening and the extracellular vesicles (EV) visualization assay on the RF-127 surface. We recently reported that SiMPull-based antibody binding assay offers a new approach to characterize antibody affinity to protein aggregates.^1^ We characterized the affinity of various *anti*-α-syn antibodies against recombinant and serum samples. Our results demonstrate that the RF-127 surface is compatible with a range of antibodies and able to quantify their binding affinity to a range of samples. In our tests, MJFR14 and Syn211 antibodies have high affinity to recombinant α-syn aggregates while LB509 shows low affinity. LB509 and Syn211 antibodies have high affinity against serum α-syn aggregates while MJFR14 shows low affinity. An IgG control condition was included to ensure the binding was specific. We also used the RF-127 surface to immobilize and visualize exosomes from human plasma. The diagnostic potential of exosomes has been extensively reported. Spitzberg et al. have recently developed a single-molecule assay to visualize EV purified from human plasma and demonstrate the diagnostic potential of this assay.^23^ We successfully captured and visualized exosomes with RF-127 surfaces. As shown in Figure 4D, we used an exosome isolation kit to separate human plasma into a high- and a low-EV portion. We only detected a high fluorescent signal in the high-EV potion with both capture and detection antibodies. All other conditions were not able to generate sufficient signals. These results indicate that the RF-127 surface is able to serve as the imaging surface in single-molecule EV visualization assays.

### Functionalizing Microplates for High-Throughput Single-Molecule Experiments

Finally, we demonstrate the feasibility of conducting robot-assisted high-throughput single-molecule experiments. Although single-molecule sensing offers a more informative sample characterization than bulk measurements, the throughput of these measurements is usually not sufficient for large-scale investigations. The initial SiMPull assay, for example, could only characterize 4 samples per plate.^20^ Super-resolution microscopy is similarly limited. Much of the current development in increased throughput focuses on the microscopy methodology, such as faster data acquisition and a larger field of view.^35,36^ There is currently very little consideration about how to utilize super-resolution microscopy for large-scale biological screening. Faster data acquisition from microscopy is not sufficient as the sample processing rate is also limited by the lengthy preparation and loading. Therefore, there is an unmet need to improve sample number throughput with a single assay. Although robot-assisted systems have been reported for biological and clinical research,^37^ they have not been applied to screen samples with SMFM on a large scale. This coating technique might be able to bridge high-throughput experiments and SMFM-based single-molecule sensing detection: Combined with a robot-assisted liquid handling system, the RF-127 surface offers an approach to improve the throughput of these experiments significantly. The functionalized microplates allow hundreds of samples to be screened on a single day. Moreover, the automated assay preparation further reduces human error during experiments as well as the need for intensive labor.

**Figure 5:**
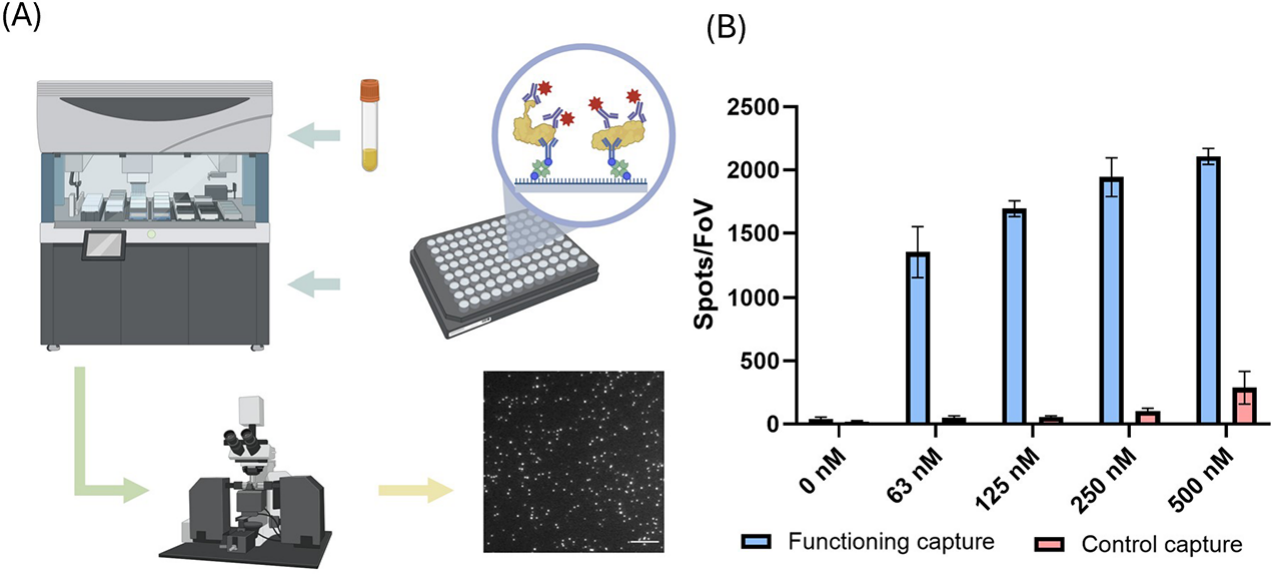
Schematic demonstration of automated SiMPull assay and detection of recombinant α-syn aggregates: (A) A 96-well plate was selectively passivated using a pipetting robot and antibodies and samples were added before being imaged. Scale bar: 10 μm. (B) Recombinant α-syn aggregates at different concentrations were tested on the automated platform. Positive results (in blue) denote the case where the correct capture/detection antibody is used. Capture control (in red) denotes the case where the capture antibody is absent, as mentioned in Figure 4A. The signal from the capture control represents the nonspecific sample–surface interaction. The concentration of aggregates is in monomer-equivalents. Each FoV contains an area of 2500 μm^2^. Error bars: s.d. (*n* = 27 from 3 experiments). No statistical tests are performed as controls and positives have more than a 10-fold difference.

We performed a semiautomated SiMPull assay with recombinant α-syn aggregates to demonstrate the potential of conducting RF-127 surface-based high throughput single molecule experiments. Most repeating steps are automatically performed apart from adding samples/probes. As shown in Figure 5, the functionalized 96-well microplate produced a clear positive correlation between sample concentration and fluorescent single-molecule counting, demonstrating a reliable quantification of recombinant α-syn aggregates. These results demonstrate the potential of developing a high-throughput single-molecule sensing platform which can perform single-molecule studies on biological samples on a large scale.

## Conclusion

To summarize, we report a much-simplified RF-127 surface for single-molecule imaging with improved passivation. In comparison to the commonly used PEG surfaces, the RF-127 surface is greener, easier to fabricate and more adaptable for use in microplates. It also has controllable capture affinity and is compatible with single-molecule techniques including SiMPull, dSTORM and DNA-PAINT. The reduced nonspecific background opens up new diagnostic applications analyzing aggregates in human biofluids.

Overall, our study offers a simple but versatile approach for surface-based single-molecule imaging, with the potential for high-throughput implementation. It can be easily produced in laboratories with limited chemistry expertise or facilities and lowers the technical barrier for relevant research.

## Methods

Please refer to the Supporting Information for additional details.

### RF127 Surface Preparation

Glass coverslips were initially cleaned using either method A or method B, where

Method A: Argon plasma clean (PDC-002, Harrick Plasma) for 10 min
Method B: Bath sonication with MQ water for 10 min and then Ethanol for 10 min, dry with nitrogen flow or compressed air can.
Method B was only used for Figure S9. All other figures in this work are based on method A.

Once glass coverslips were cleaned, a PDMS gasket (Sigma, GBL103250–10EA) was attached to the surface. Six μL of coating solution (Mixture of Rain-X and isopropanol using 1:1 ratio and filtered using a 200 nm filter) was loaded into each well. Critically, the coating solution must be passed through a PVDF filter (Millex, SLGV004SL) prior to use. Filters with other membrane materials (like PES) or different manufacturing processes (nonsterilized) will significantly comprise the results under tested conditions. A large residue may appear if an incompatible filter is used (see Figure S12 in Supporting Information). The coating buffer was left to dry naturally. Extra rinsing with filtered isopropanol may be applied to further clean up if coating solution residues remain. The coated coverslip can be stored at room temperature for 2 weeks. The coating can last up to a few months, but we recommend using it within 2 weeks to prevent bacterial contamination. Rain-X (Rain-X Rain Repellent 200 mL) used in work was purchased from a local store (Halfords, Cambridge, UK, CB5 8WR). NeutrAvidin and F-127 coating will be applied during the phase of assay preparation. Details can be found in the section Single-molecule pull-down experiment.

### Passivation Test of Coverslips

The coated coverslip was then rinsed 2× with PBS by pipetting PBS in and out of the wells, before being incubated with NeutrAvidin solutions (0.1 mg/mL) for 15 min and washed 3× with PBST. Optional BSA (R&D Systems, DY995) blocking can be performed after this step (1% BSA in PBST for 20 min, with 2× PBST washing at the end). This step was only included in the passivation test for the PEG surface. Proteins at different concentrations were loaded on a coverslip for 10 min and washed 3× with PBST. Relevant detection antibodies (500 pM in PBST) were then loaded on coverslips for 5 min and washed 3× with PBST.

### Single-Molecule Pull-Down Experiment

#### Preparation Stage (RF-127 and PEG Surface has Different Protocols)

For RF-127 surface: The coated coverslip needs to be rinsed 2× with PBS by pipetting PBS in and out of the wells, then incubated with NeutrAvidin solution with the desired concentration and incubation time. We used 0.1 mg/mL for 15 min as the default setting. However, as discussed in the main text, this step can be modified to adjust surface capture affinity. Once NeutrAvidin incubation was complete, the wells were rinsed 3× with PBS by pipetting PBS in and out again. 1% F-127 solution (Invitrogen, P6866), made by mixing 10% stock with PBS and passing through a 200 nm filter, was loaded into the wells and incubated for 45 min. F-127 residues were then washed by rinsing the coverslip 3× using PBST. Where necessary, a BSA blocking step (1% BSA in PBST and incubated for 20 min, with 2× PBST washing at the end) can be performed at this point. This step was performed for all RF-127 based SiMPull experiments.

For the PEG surface: 0.1 mg/mL NeutrAvidin was incubated for 15 min and rinsed 3× with PBST by pipetting PBS in and out again.

#### Sample Characterization Stage (RF-127 and PEG Surface has Same Protocol)

Then 10 nM of relevant biotinylated capture antibodies were diluted in the PBST and incubated in each well for 5 min. After incubation, coverslips were rinsed 3× with PBST. PBST was incubated for around 30 s during the washing process. Samples should then be loaded onto wells. Unless specifically mentioned, we incubated recombinant proteins for 45 min (Figure 1), 15 min (Figures 5, S1, S3, S8, S9), biofluids (serum, CSF, saliva and exosomes) for 90 min, and brain extracts for 150 min. Once the sample incubation is complete, coverslips require rinsing 3× with PBST before incubation with detection antibodies. For recombinant proteins, we incubated with detection antibodies at 500 pM for 5 min. For other biological samples, we used 5 nM for 20 min. Following detection antibody incubation, coverslips were once again washed 3× with PBST. Before imaging, wells were filled with either PBS (for diffraction-limited imaging), STORM buffer (for dSTORM imaging, 2 mg/mL glucose oxidase (Sigma, G7141–250 KU), 52 μg/mL catalase (Sigma, C3515) and 7 mg/mL cysteamine (Sigma, M9768–5G) at pH 8.0 in Tris-PBS containing 10% glucose), or DNA imager strands (for DNA-paint imaging, 2 nM imager in PBS). The imaging strand (ATGTAGAT) was conjugated with Cy3B. SiMPull-based antibody screening assay follows the same protocol.

### Microscopes and Data Analysis

The setups used in this work are similar,^8^ but with slight differences:

- Set up A (used for data in Figures 1–4, S1–S8 and S10): 488 nm (iBeam- SMART, Toptica), 561 nm (Cobalt Jive, Cobalt) and 638 nm lasers (Cobolt MLD 638, Cobalt) were coupled into a TIRF objective (NA 1.49, Apo TIRF, 60XO TIRF, Olympus) mounted on an inverted Ti-E Eclipse microscope (Nikon, Japan), via a multimode optical fiber. A built-in Perfect Focus system was used to lock the focal plane when imaging. The fluorescent emission was collected by the same objective, separated from the excitation laser using a dichroic (Di01-R405/488/561/635, Semrock), and directed to an EMCCD-camera (Evolve 512, Photometrics) through a 1.5× beam expander. Emission filters (LP02-568RS25, Semrock and FF01-587/35–25, Semrock) were used to cleanup the signal. Each pixel corresponded to 102.3 nm. Data acquisition was carried out using MicroManager.
- Set up B (used for data Figures 5 and S9): 638 nm laser (Cobolt MLD 638, Cobalt) was coupled into a TIRF objective (NA 1.49, Apo TIRF, 60XO TIRF, Olympus) mounted on an inverted Ti-E Eclipse microscope (Nikon, Japan), via a free-space path. A built-in Perfect Focus system was used to lock the focal plane when imaging. The fluorescent emission was collected by the same objective, separated from the excitation laser using a dichroic (Di01-R405/488/561/635, Semrock), and directed to an EMCCD-camera (Evolve 512, Photometrics) through a 1.5× beam expander. Emission filters (LP02-568RS25, Semrock and FF01-587/35-25, Semrock) were used to clean up the signal. Each pixel corresponded to 107.0 nm. Data acquisition was carried out using MicroManager.

## Data Availability

Additional data supporting this manuscript is included within the associated Supporting Information.

## Supporting Information Available

It contains the supplementary methodology and supplementary characterization of the RF-127 surface (PDF)

## Notes

The code used for this work is available at https://github.com/YPZ858/DF-single-molecule-couting—(single-molecule counting) and https://github.com/YPZ858/Super-res-code/issues (super-resolution imaging). The exposure time per frame is 50 ms for diffraction-limited images, 15 ms for dSTORM images and 100 ms for DNA-PAINT. Briefly, single-molecule counting was performed using a single-molecule localization engine in ThunderSTORM.^38^ The images were processed using a wavelet filter and the localizations were identified using a hybrid threshold of 0.5 × std (Wave.F1) + 0.1 × mean (Med.F). This arrangement gives a higher resistance to noise by combining the adaptive threshold and a mean-value threshold. The super-resolution imaging reconstructions were done via drift correction,^39^ peak fitting and postfit analysis. The morphological analysis module in our code was not used in this work.
