# Supplementary material for: Improved Imaging Surface for Quantitative Single-Molecule Microscopy
Source: ACS Appl Mater Interfaces. 2024 Jul 9;16(28):37255–64. doi: 10.1021/acsami.4c06512 (PMC11261557; doi:10.1021/acsami.4c06512)
Supplement: Supplementary file 1 — am4c06512_si_001.pdf [file am4c06512_si_001.pdf]

Supporting Information

An improved imaging surface for quantitative single-molecule microscopy

Yu P. Zhang[a,b], Evgeniia Lobanova[a,b], Asher Dworkin[a,b], Martin Furlepa[a,c], Woo Suk Yang[a,b], Melanie Burke[a,b], Jonathan X. Meng[a], Natalie Potter[a], Renata Lang Sala[a], Lakmini Kahanawita[c], Florence Layburn[a,b], Oren A. Scherman[a], Caroline H. Williams-Gray[c] and David Klenerman[a,b] *

[a] Department of Chemistry, University of Cambridge, Lensfield Road, Cambridge CB2 1EW, United Kingdom
[b] UK Dementia Research Institute at Cambridge, Cambridge CB2 0XY, United Kingdom
[c] Department of Clinical Neurosciences, University of Cambridge, Cambridge CB2 0PY, United Kingdom

*Email: David Klenerman (dk10012@cam.ac.uk)

This supplementary material includes:

Supplementary methods
Table S1. Antibody and aptamer used in the work
Table S2. Patient information
Figure S1 Measuring autofluorescence of the RF-127 surface
Figure S2 Contrast-adjusted version of representative images.
Figure S3 Represent images of specific and non-specific binding of tau aggregates on both PEG and RF127 surfaces.
Figure S4 Comparing passivation between RF-127 surface and Sigmacote-F-127 surface.
Figure S5 Comparing capture antibody density between RF-127 surface and PEG surface.
Figure S6 Contact angle measurement of RF-127 surface.
Figure S7 Reduction of specific SiMPull signals with different washing steps.
Figure S8 The effect of Rain-X coating.
Figure S9 Stability and controllability of surface capture affinity.
Figure S10 Reproducibility of surface capture and passivation.
Figure S11 Representative single molecule images used for the super-resolution microscopy test.
Figure S12 Representative photo of good and defective coating.

Supplementary methods

Buffer, Antibodies and aptamers

This work used PBS (Gibco™, 10010015) buffer system. PBST (PBS with 0.05%) was made by diluting commercial PBST stock (Rockland, MB-075-1000). The aptamers used in experiments were purchased from ATDbio, with 2X HPLC purification. The antibodies used for diffraction-limited and dSTORM imaging were purchased commercially. Details of these antibodies can be found in Table S1.

PEG surface preparation

Coverslips were prepared following a previous report.[2] Glass coverslips (26x76 mm, thickness #1.5, VWR, Cat. No. MENZBC026076AC40) were sequentially cleaned with bath sonicator (Ultrasonic cleaner USC100T, VWR) using MQ water (18.2 -MΩ cm−1), acetone, and methanol. Each step lasts for 10 minutes. Cleaned coverslips were then etched by 1M KOH for 20 min with sonication under the same condition as previous steps. The etched coverslip was rinsed with MQ water and then methanol. Methanol residues on the glass were removed by nitrogen flow. The dried coverslips were then cleaned with argon plasma for 15 min (Femto Plasma Cleaner; Diener Electronic) and silanized with 5 ml of 3-aminopropyl triethoxysilane (Fisher Scientific UK, cat. no. 10677502), 8.3 ml of Acetic acid in 166 ml of methanol for 20 min. During the process, the reaction chamber was bath sonicated for 1 minute at the beginning and midpoint (10 min from the start). The salinized coverslips were then sequentially rinsed by methanol, MQ water, methanol again to remove the residue from the previous step, and then dried with nitrogen flow. 50-well polydimethylsiloxane (PDMS) chamber gasket (cut from a CultureWell chambered coverglass, Sigma, cat. no. GBL103350-20EA) were attached to the coverslips. PEGylation of coverslip was performed in the wells by attachment of gasket. Each well was treated with 9 µl of a 100:1 aqueous mixture of methoxy-(110 mg/ml, ~22 mM, Mw ~5,000; Laysan Bio Inc., cat. no. MPEG-SVA-5000) and biotin-terminated (1.1 mg/ml, ~220 µM, Mw ~5,000, Laysan Bio Inc., cat. no. Biotin-PEG-SVA-5000) PEGs. 1 µl of 1 M NaHCO3 (pH 8.3) was loaded after the PEG solution to speed up the reaction. It needs to be mentioned that mixing $NaHCO_3$ with PEGs at the first step may accelerate the hydrolysis and reduce the coating efficiency, therefore we add the $NaHCO_3$ after PEG. The reaction was performed at room temperature overnight in a humid chamber. Coated coverslips were then rinsed with MQ water and dried with Nitrogen flow. A second-round PEGlytion was then performed to enhance the passivation. 9 µl of smaller methoxy-terminated PEG (10 mg/ml, 30 mM, MS(PEG)4 methyl-PEG-NHS-Ester; MW~333, ThermoFisher, cat. no. 22341) was added to each well before further loading of 1 µl of 1 M $NaHCO_3$ (pH 8.3). The reaction was done at room temperature overnight in a humid chamber. Coated coverslips were then rinsed with MQ water and dried with Nitrogen flow. Coverslips were stored in a desiccator at −20 °C until use.

Sigmacote surface preparation

The glass coverslip was initially cleaned with Argon plasma for 10 min and then incubated within Sigmacote (a silicone solution, Sigma, SL2) for 5 min at room temperature. A designated glass incubation chamber was used in this step to avoid potential contamination. The coated glass was air-dried for 30 minutes. The F127 coating step is identical to the RF-127 method. (1% F-127 in PBS for 45 minutes).

Microplate passivation

30μl of coating solution (see previous section for details) was loaded into each well (Corning, 4680, Cyclic Olefin Copolymer) for 10 min and removed by aspiration pump (VACUSAFE, Integra). The treated microplate was then left to dry on a bench for 30 min-1 h (until completely dried). The storage condition for the microplate was the same as the coverslip.

Passivation test of coverslips

The coated coverslip was then rinsed 2X with PBS by pipetting PBS in and out of the wells, before being incubated with NeutrAvidin solutions (0.1 mg/ml) for 15 min and washed 3X with PBST. Optional BSA (R&D Systems, DY995) blocking can be performed after this step (1% BSA in PBST for 20 min, with 2X PBST washing at the end). This step was only included in the passivation test for PEG surface. Proteins at different concentrations were loaded on a coverslip for 10 min and washed 3X with PBST. Relevant detection antibodies (500 pM in PBST) were then loaded on coverslips for 5min and washed 3X with PBST.

Robot-assisted automation

Assay automation was performed using the CyBio FeliX liquid-handling robot (Analytik Jena) according to the manufacturer's instructions.
The assay preparation is largely the same as the previous section *of the Single-molecule pull-down experiment*, with a few exceptions: (1) the imaging well was filled with 40 μL liquid while 10 μL was applied on the coverslip. (2) 4 cycles of washing were performed on the robotic system while 3 were on the coverslip. Each cycle contains 5 aspirations. (3) The sample removal and washing steps were performed by the automated system, while NeutrAvidin, samples and antibody loading were done manually. The antibody/material used is identical to the conditions used for recombinant α-syn aggregates in Figure 1 and 2.

Sample preparation

Aggregation of α-syn: Monomeric wild-type α-syn was obtained from Escherichia coli (*E. Coli*) according to an existing protocol. [1] 70 μM of these monomers were supplemented with 0.01% NaN_3 in PBS and incubated for 96 h with constant shaking at 200 rpm at 37 °C. Sonicated α-syn aggregates were produced with pulse sonication (QSonica, Q125, tip size 1/8 in.). Each pulse consisted of a 5s on state and a 15 sec off state at 40% power. 3 cycles were performed.

Aggregation of Aβ: Monomeric $A\beta_{1\text{-}42}$ was purchased commercially (Bio Trend, AS-24235) and purified according to the supplier's protocol. 20 μM of these monomers were supplemented with 0.01% NaN_3 in 20 mM NaP 10 mM NaCl 180 μM EDTA and incubated for 48 h at 37 °C. Sonicated Aβ aggregates were produced with pulse sonication (QSonica, Q125, tip size 1/8 in.). Each pulse consisted of a 5 sec on state and a 15 sec off state at 40% power. 3 cycles were performed.

Aggregation of tau: Monomeric tau was obtained according to an existing protocol[3]. Briefly, full-length tau (isoform 0N4R) wild-type were transformed in BL21(DE3) cells and harvested from lysates. 2 μM of these monomers were supplemented with 0.01% NaN_3 in PBS and incubated for 72h at 37 °C.

Aggregation of p53: Monomeric wild-type p53 aggregates were prepared following an existing protocol [4]. Briefly, p53 protein samples were expressed and purified from insect cells by GenScript Biotech. Monomeric wild-type p53 was diluted to 100 nM with the aggregation buffer (5mM DTT, 50mM Tris, 150mM NaCl, pH 7.2) and incubated for 72h with constant shaking at 200 rpm at 37 °C. The aggregates were sonicated with an ultrasonic cleaner (VWR, USC-T) for 5min with ice before use.

Human serum, plasma and CSF: Serum, plasma and CSF samples were collected from patients with idiopathic PD. They were participants in a University of Cambridge, UK study (NET-PDD: Neuroinflammation and Tau Aggregation in Parkinson's Disease Dementia). All participants provided informed consent and ethical approval was given by the East of England-Essex Research Ethics Committee (16/EE/0445). Blood was collected from participants using venepuncture with S-Monovette tubes (7.5 ml). Collected samples were left to clot for 15 min at room temperature and centrifuged at 2000 rpm for 15 min. The supernatant (serum) was obtained and stored at -80 ℃. For plasma sample, blood was collected from participants using 9 mL S-Monovette tubes (Sarstedt, 02.1066.001) and centrifuged at 600 g for 15 min at room temperature. The supernatant (the plasma) was then collected and stored in 500 mL aliquots at -80 °C until further use. CSF was collected from participants via lumbar puncture under sterile technique with 1% lidocaine as local anaesthetic. Collected samples were centrifuged at 300x g for 10 min at 4 ℃. The supernatant was stored at -80 ℃.

Human saliva: Saliva samples were collected from idiopathic Parkinson's disease patients recruited via the Cambridge Parkinson's Research Clinic (University of Cambridge, UK). Healthy age-matched controls were recruited from the same centre. Ethical approval was given by the East of England – Central Cambridge Research Ethics Committee (03/303). All participants provided written informed consent. Participants were required to fast for at least 1 hour prior to sample collection. Exclusion criteria included ongoing dental or oral disease, smoking within four hours prior to sample collection, and consuming alcohol within 12 hours of sample collection. Samples were collected between 10 and 11:30 am using the passive drool method. Patients were instructed to allow saliva to pool in their mouths and drool into a sterile container. No stimulation was applied. All samples were collected and processed over ice. After collection protease inhibitors (Sigma P, x10 concentration, 10 µL per ml of saliva) and phosphatase inhibitors (sodium orthovanadate 3 µL per ml of saliva) were added. Samples were then centrifuged at 2600xg at 4 ˚C for 15 min. The supernatant was collected and underwent a further 15000x g centrifuge step at 4 ˚C for 15 min. The resulting supernatant was then removed and stored in 100 µL aliquots in cryotubes at -80 ˚C. Received 100 µL aliquots were then defrosted and divided into smaller 11 µL aliquots before being stored at -80 ˚C for further analysis. There were no further freeze-thaw cycles.

Post-mortem brain: Post-mortem brain tissue was acquired from the Cambridge Brain Bank (with the approval of the London—Bloomsbury Research Ethics Committee; 16/LO/0508, Table 1). The brain samples have been voluntarily donated without any compensation. Brains were flash-frozen and stored at −80 °C. Fresh-frozen brain tissue was homogenised (the brain sample H in the main text) using a method adapted from literature[5]. Briefly, the tissue was homogenised at 4 °C in a VelociRuptor V2 Microtube Homogeniser (Scientific Laboratory Supplies, Cat. No. SLS1401) in 10 volumes of homogenisation buffer (10mM Tris-HCl, 0.8 M NaCl, 1 mM EGTA, 0.1% Sarkosyl, 10% sucrose; pH 7.4) containing complete™ Protease Inhibitor and PhosStop™ Phosphatase Inhibitor. The homogenate was centrifuged at 21,000x g for 20 min at 4°C, and the upper 90% of the supernatant was retained. An additional 5 volumes of homogenisation buffer was added to the pellet, and after another round of homogenisation and centrifugation, the upper 90% of this supernatant was combined with the first supernatant, and this mixture was aliquoted and frozen at -80 °C until used for experiments. This sample was diluted in ELISA diluent (ab193972) with a 1:3 ratio when performing single-molecule experiments. Soaked brain extracts (The brain sample S in the main text) were prepared from 300 mg of flashed-frozen (stored at −80 °C) tissue from the amygdala. This was placed into 1.5 mL of artificial cerebrospinal fluid buffer (aCSF, 124 mM NaCl, 2.8 mM KCl, 1.25 mM NaH2PO4, 26 mM NaHCO3; pH 7.4, supplemented with 5 mM EDTA, 1 mM EGTA, 5 µg/mL leupeptin, 5 µg/mL aprotinin, 2 µg/mL pepstatin, 20 µg/mL Pefabloc, 5 mM NaF) for 30 min at 4 ˚C and centrifuged at 2000x g for 10 min. The supernatant (~90%) was transferred into a fresh tube and centrifuged at 14,000x g for 2 h. After the centrifugation, supernatant (~90%) was dialysed for 72 h using Slide-A-Lyzer cassettes (MKCO 2kDa, Thermo Scientific, Cat. 66330). Three buffer exchanges against aCSF at 4˚C were

performed during the process. The product was stored at -80 ℃. This sample was diluted in ELISA diluent (ab193972) with a 1:1 ratio when performing single-molecule experiments.

EV purification from human plasma
Human plasma was processed using EXO-Prep exosome isolation kit (HBM-EXP-B10), following the manufacturer's protocol. The effluent (low exosome fraction) was kept for control experiments.

DNA-antibody conjugation for DNA-PAINT
DNA-antibody conjugation was generated using an existing protocol[6], with minor modifications. Briefly, antibodies were purified and concentrated using a PBS-rinsed amicon ultracentrifuge filter (Millipore, UFC5100). 3 buffer exchange cycles were performed. Concentrated antibodies were then incubated with DMSO-dissolved DBCO-PEG$_4$-NHS (Sigma, 764019) for 3 h. The products were purified using amicon ultracentrifuge filters. 3 buffer exchange cycles were performed. The DBCO-modified antibodies were then incubated with azide-modified DNA docking strand (TACATCTA, ATDbio) for 3h. The products were purified using amicon ultracentrifuge filters again with 3 buffer exchange cycles. The concentration of antibodies as well as DNA labelling efficiency was characterised using Nanodrop 2000. The stock is stored at 4 ˚C until use.

Dye-labelling and biotinylation of antibodies
We used a commercial kit for dye labelling (Invitrogen, Z11235) and biotinlytion (Thermo, 90407). The processing was completed according to the manufacture's protocol with 2 additional purification steps. Plain antibodies were purified using Amicon filters with 3 buffer exchange cycles before processing. The subsequent products were then purified using the same method. The purification method was the same as that used in DNA-antibody conjugation, detailed above.

Contact angle measurement
The contact angle was measured using a FTA1000 Drop Shape Instrument B Frame System with an instrument tilt and motorized syringe control from First Ten Angstroms. Images and video were recorded using a Navitar camera. The MilliQ water droplets were deposited onto the slides from a 100 µL glass syringe (internal diameter 1.400 mm) with a steel plunger from Hamilton company. The First Ten Angstrom 32 software was used to start the droplet formation, record, and measure the contact angle. 3 images were taken before the trigger and 25 images were taken post-drop to evaluate any changes to the droplet contact angle over time. Contact angles were measured immediately after droplet made full contact with the slide and 3-4 different positions on each slide were measured and then averaged.

Table S1. Antibody and aptamer used in the work

Data in	Item	Information
Figure 2,3	AF647 IgG antibody	MOPC-21 ,AB_2539542, Invitrogen
Figure 2, S7	AF647 DNA aptamer (T-SO508)	AF647-T-SO508, ATDbio
Figure4(A),4(C),5,S7,S8,S9, S10	AF647 α-syn antibody(211)	sc-12767 AF647,santa cruz biotech
Figure 2	AF647Aβ antibody(6E10)	803020,biolegend
Figure 2, 4(A),S3,S4	AF647 tau antibody(HT7)*	MN1000,Invitrogen, with modification
Figure 2	AF647 p53 antibody	sc-12767 AF647,santa cruz biotech

Figure4(A),4(C),5,S1,S5,S7,S8, S9,S10	Biotin α-syn antibody(211)*	sc-12767, santa cruz biotech, with modification
Figure4(A),S3	Biotin tau antibody(HT7)	MN1000B,Invitrogen
Figure4(B)	Biotin α-syn antibody(211)*	sc-1276, santa cruz biotech, with modification
Figure4(B),S10	DNA-labelled α-syn antibody(211)*	sc-1276, santa cruz biotech, with modification
Figure4(C),S1,S5	Biotin α-syn antibody(MJFR14)	ab216309, abcam
Figure4(C)	AF647 α-syn antibody(MJFR14)	ab227047, abcam
FigureS1	α-syn antibody(211)*	sc-12767, santa cruz biotech
FigureS1,S5	AF647 anti-mouse IgG	ab150115
FigureS1	AF568 anti-mouse IgG	ab175473
FigureS1	AF488 anti-mouse IgG	ab150105
Figure4(C)	Biotin α-syn antibody(LB509)	807710, biolegend
Figure4(C)	AF647 α-syn antibody(LB509)	sc-58480 AF647
Figure4(D)	AF647 CD81 Antibody	FAB4615B
Figure4(D)	Biotin CD63 Antibody	A85980

*Modifications including dye labelling and biotinylation

Table S2. Patient information

Sample type	Gender	Age	PD duration (years)	updrs_total	Cause of death
Serum	F	56.28	0.62	44.00	N/A
Plasma	F	62.9	0.47	110	N/A
CSF	M	69.8	1.67	53	N/A
saliva	F	69.88	4.73	52	N/A
Brain (S&H)	M	85.89	18.11	N/A	Malignant Neoplasm of ascending Colon, PD

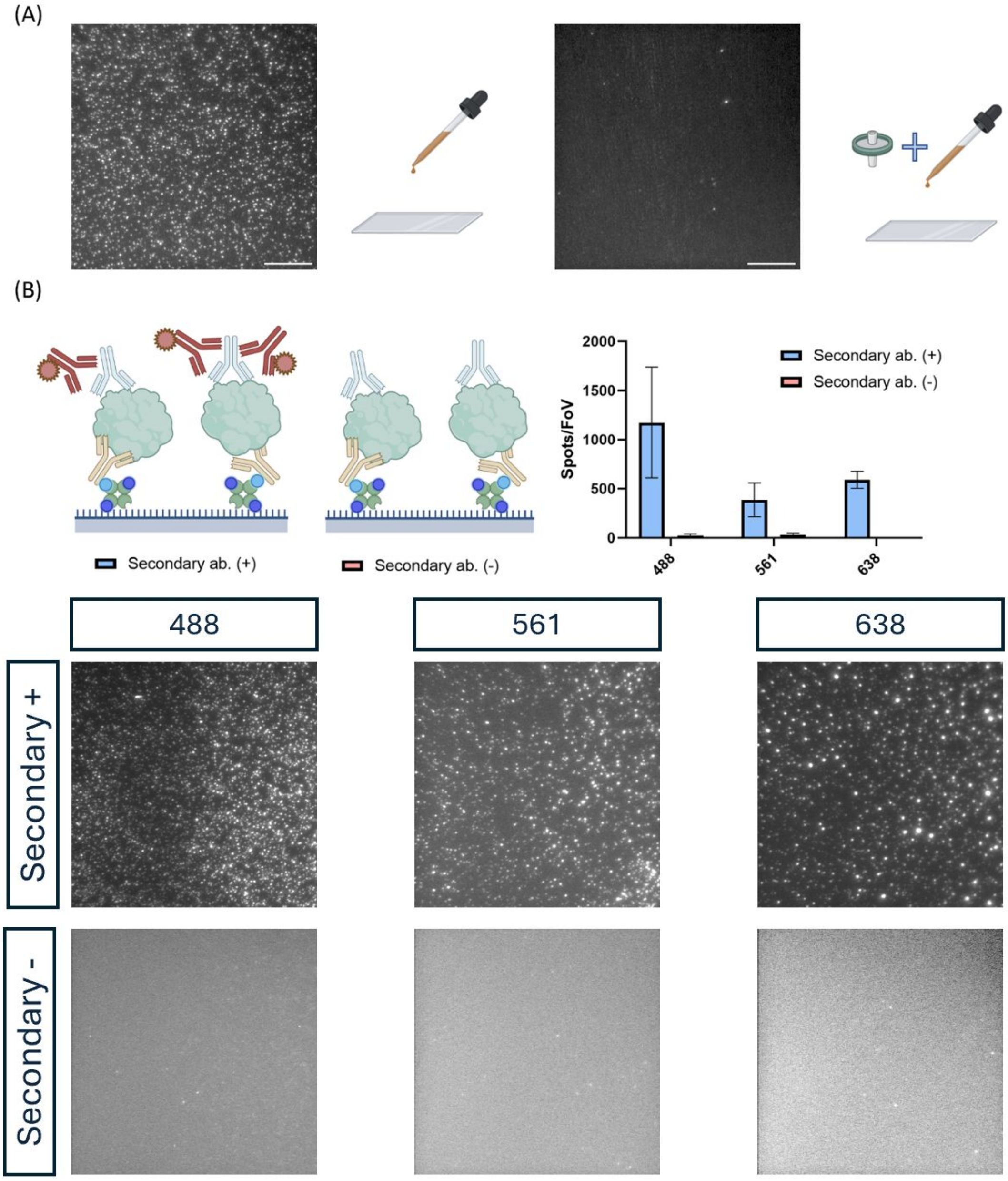

Figure S1 Measuring autofluorescence of the RF-127 surface **(A)** The surface exhibits high-level autofluorescence (561 nm laser line only) when reagents are not filtered (shown on the left panel). On the other hand, filtering can effectively eliminate these backgrounds RF-127 surface has low autofluorescence when made using filtered Rain-X coating buffer and F-127 solution (right panel). Scale bar: 10µm **(B)** Properly prepared RF-127 surface possesses a low level of autofluorescence. With the absence of a fluorescent probe (red data bars), the sample-loaded RF-127 surface generated a very low signal. The signal only appears when a relevant probe is added (blue data bars). To avoid the cross-talk of primary and secondary antibodies, the MJFR14 (Rabit) antibody was used for capture, and Syn211 (Mouse) was used for visualisation. Fluorescently labelled anti-mouse secondary antibodies are used for visualisation (See Table S1 for details). Recombinant α-syn aggregates were used for this test. The contrast of the images had been adjusted to make all single molecules visible (gamma = 0.5). Each FoV contains an area of ~2500 µm^2. Error bars, s.d. (n = 16).

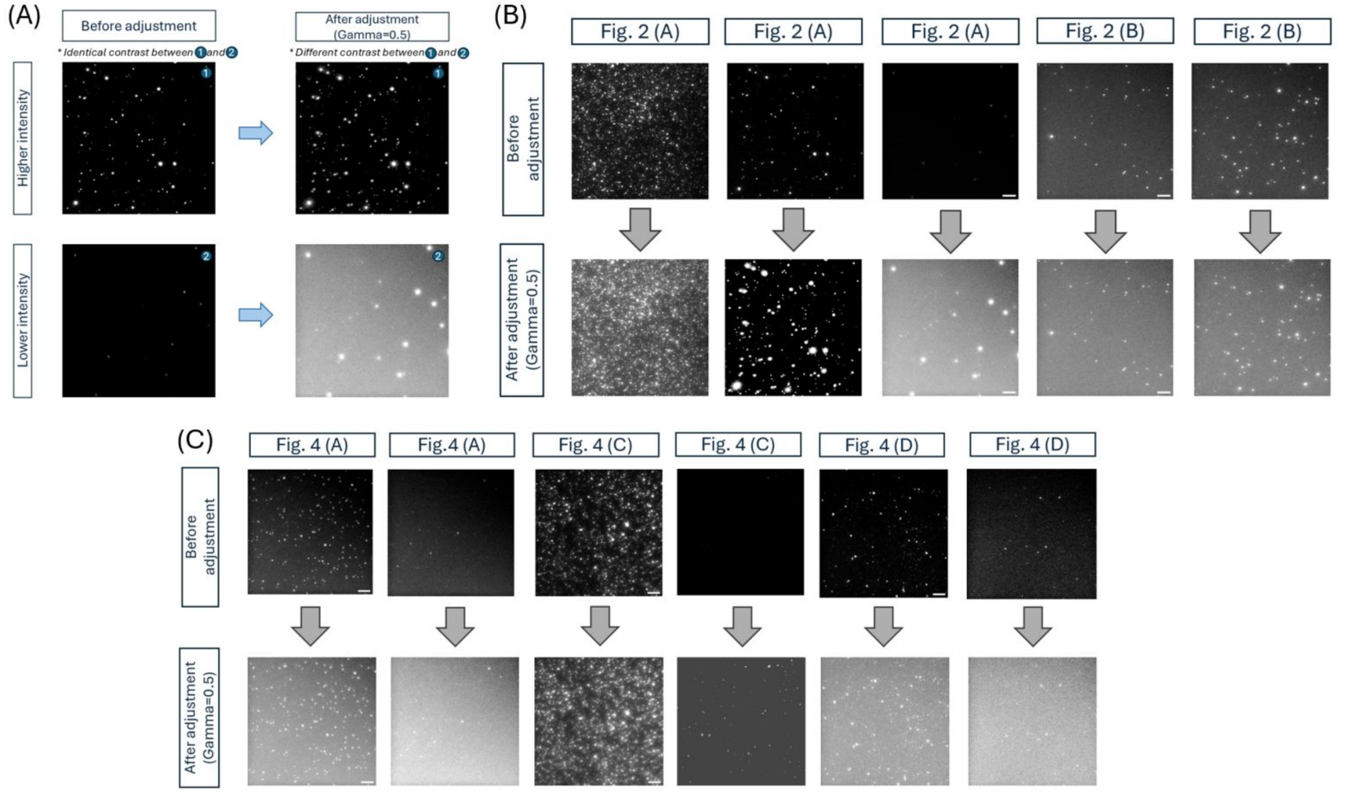

Figure S2 Contrast-adjusted version of representative images. **(A).** When the identical brightness setting was applied, paired images with very different raw brightness may make molecules invisible. Adjusted contrast (gamma = 0.5) can make all detected single molecules visible. **(B)** and **(C)** Adjusted versions of representative images included in the paired comparison within the main text. Each FoV contains an area of ~2500 µm^2.

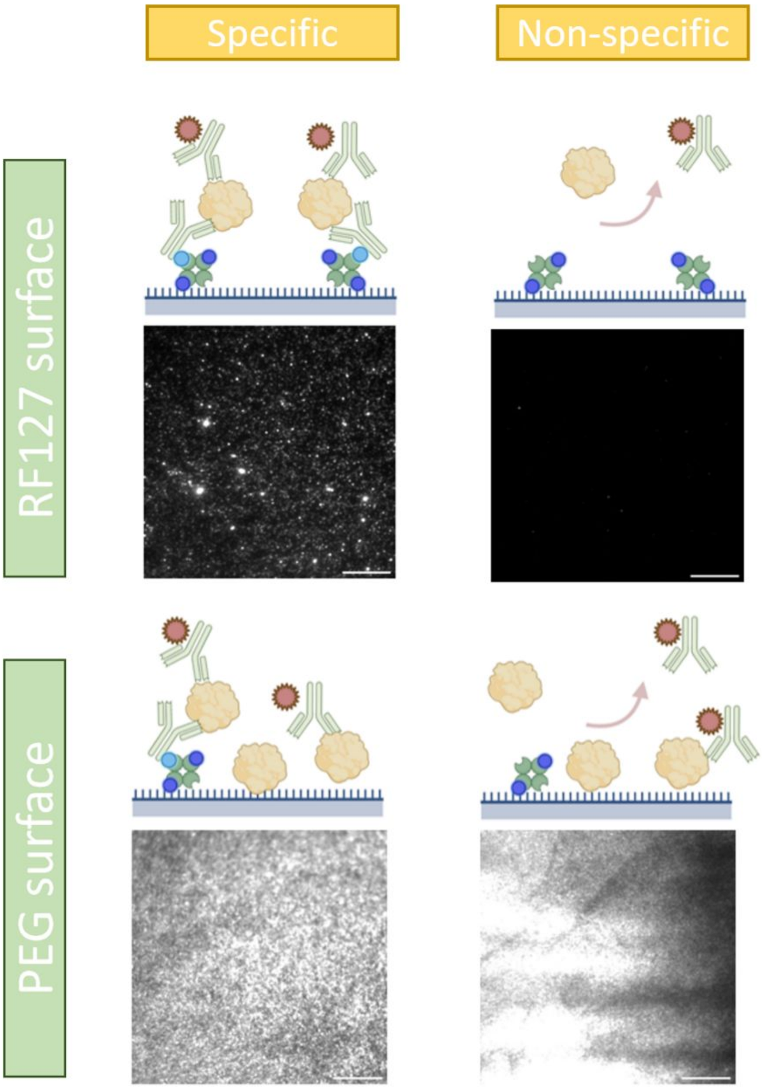

Figure S3 Represent images of specific and non-specific binding of tau aggregates on both PEG (lower) and RF127 (upper) surfaces. The specific binding is achieved by the capture antibodies coated on the surface (left), the non-specific binding is caused by the direct non-specific interaction between the surface and the aggregates(right). PEG surface has a high-level non-specific binding and is unable to generate analysable single-molecule images. Each FoV contains an area of ~2500 µm^2. Scale bar: 10µm.

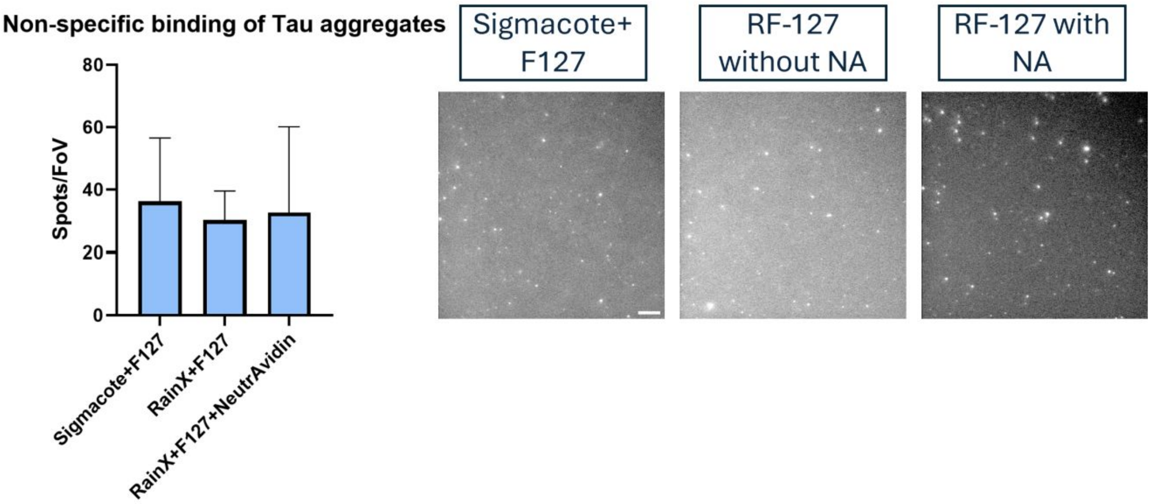

Figure S4 Comparing passivation between RF-127 surface and Sigmacote-F-127 surface. The level of non-specific binding was quantified by counting the number of non-specific absorbed tau aggregates. (200 nM). Little differences were found between the two methods. The deposition of NeutrAvidin did not alter the surface passivation in this test. Error bars, s.d. (n = 9). Scale bar: 5 µm.

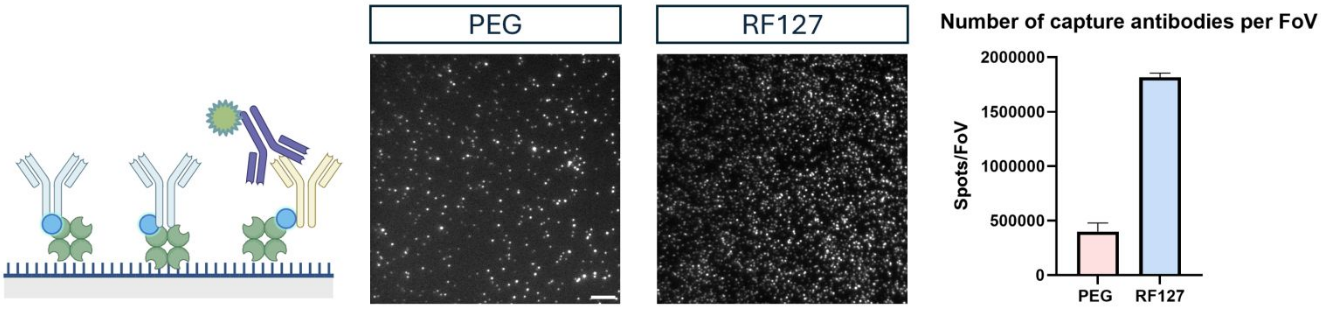

Figure S5 Comparing capture antibody density between RF-127 surface and PEG surface. Biotinylated rabbit antibody (MJFR14) was mixed with biotinylated mouse antibody (syn-211) at a ratio of 1000:1. Fluorescent secondary antibody (AF-647 anti-mouse) was used to quantify the number of immobilised mouse antibodies. The capture antibody density was estimated by multiplying the mouse antibody numbers by 1000. Scale bar: 5 µm.

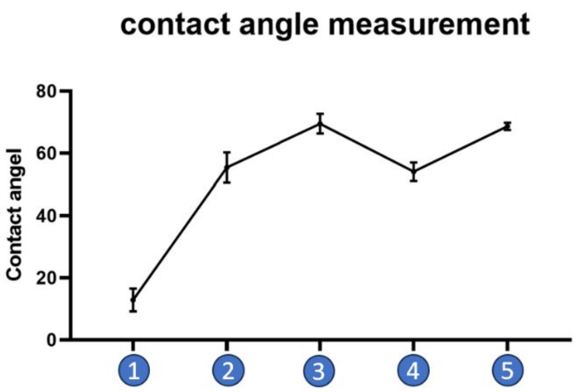

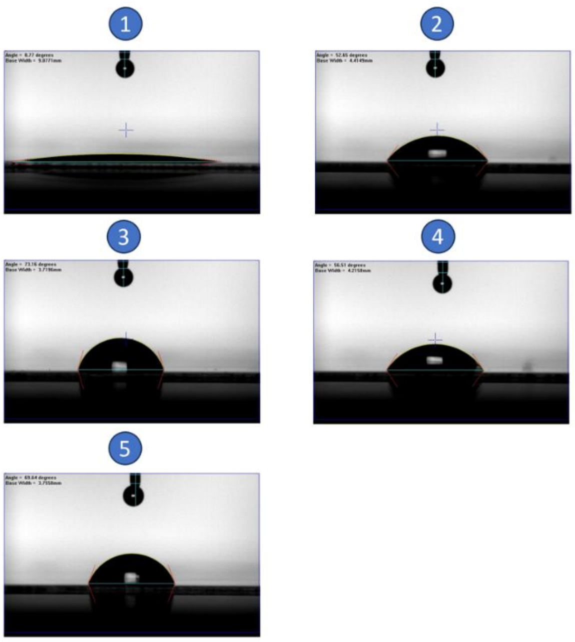

	1	2	3	4	5
Plasma cleaning	+	+	+	+	+
Rain-X coating	-	+	+	+	+
NeutrAvidin coat	-	-	+	+	+
F-127 coating	-	-	-	+	+
BSA coating	-	-	-	-	+

Figure S6 Contact angle measurement of RF-127 surface. The contact angle data varies throughout the various coating steps, detailed above. Note these measurements were conducted on dried surfaces, whereas the surfaces remained wet during assay preparation. Hence, the contact angle measurements are more suitable for providing a semi-quantitative interpretation of surface modification. Error bar s.d.= 3.

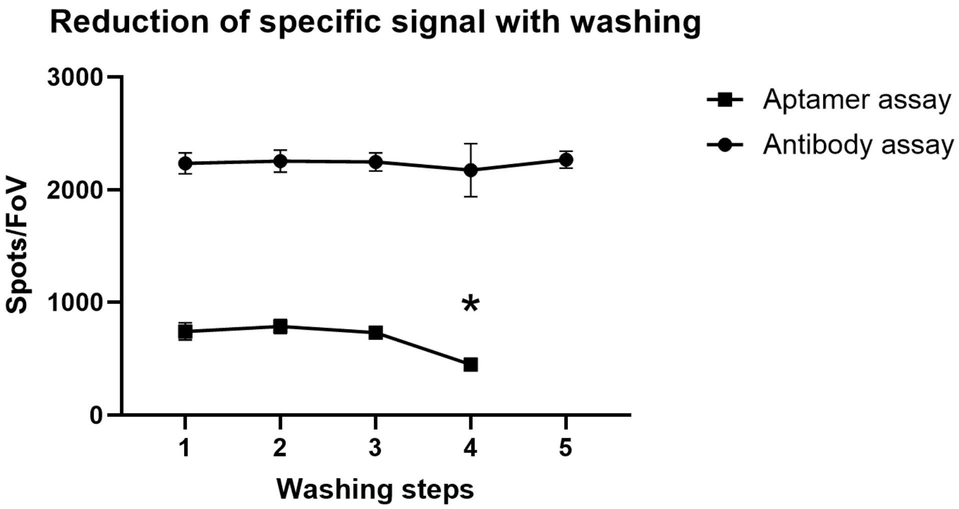

Figure S7 Reduction of specific SiMPull signals with different washing steps. SiMPull imaging was employed to assess the impact of various washing steps on specific signals. Utilizing biotin-syn-211 antibody as the capture agent and AF647-syn-211 antibody/T-SO508 aptamer as the detection agent for α-syn aggregates in human serum, no substantial reduction in signal was observed for syn-211 assay. T-SO508 assay started to have a significant signal loss after 4 washes. The Syn-211 affinity (K_d < 41 nM) [7] is higher than T-SO508 (K_d = 68 nM) [8] Error bars, s.d. (n = 16). One-way ANOVA was used to statistical test. * denotes p<0.05.

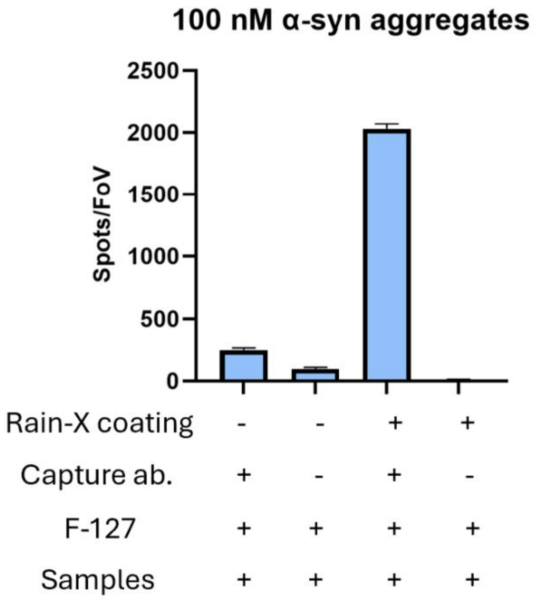

Figure S8 The effect of Rain-X coating. Rain-X coating changes the hydrophobicity of the surface and enables sufficient binding of neutravidin and F-127 layer. When surface is not coated by Rain-X, it has relatively poor capture affinity and a higher level of non-specific binding. Error bars, s.d. (n = 9).

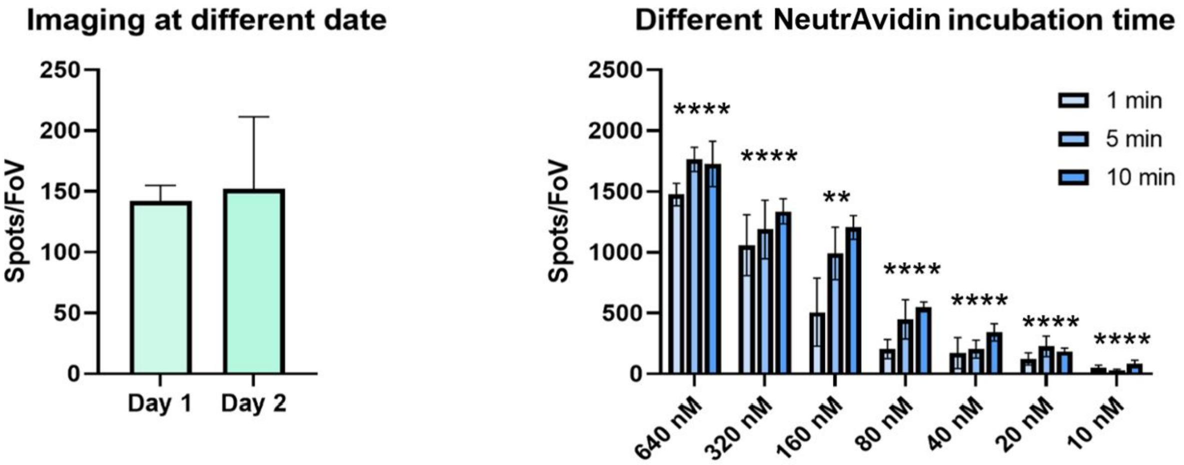

	1 min		5 min		10 min		ANOVA summary			
	Mean	std.	Mean	std.	Mean	std.	F	P value	P summary	R squared
640 nM	1475.44	89.39	1764.06	95.81	1727.50	180.61	22.32	<0.0001	****	0.4980
320 nM	1059.94	242.08	1187.56	233.54	1337.38	99.76	45.45	<0.0001	****	0.6689
160 nM	507.75	271.16	991.12	209.08	1205.19	95.63	7.05	0.0022	**	0.2386
80 nM	205.50	77.03	450.38	156.24	551.38	39.58	44.61	<0.0001	****	0.6647
40 nM	172.50	122.69	204.81	71.26	342.25	68.08	14.76	<0.0001	****	0.3962
20 nM	123.81	48.88	227.13	81.28	187.25	25.50	12.66	<0.0001	****	0.3602
10 nM	51.19	20.02	27.69	10.29	81.81	29.85	23.71	<0.0001	****	0.5131

Figure S9 Stability and controllability of surface capture affinity. Left: The fluorescent signal of α-syn aggregates remains the same on the coverslip after 24 h. Right: the surface capture affinity can be controlled by modifying NeutrAvidin incubation time. Longer incubation will yield a higher density of capture antibodies and hence improve the capture affinity of recombinant α-syn aggregates. Each field of view contains an area of 2500 μm^2. Error bars, s.d. (n = 16). The table below summarises the statics of the data shown upright (different NeutrAvidin incubation time).

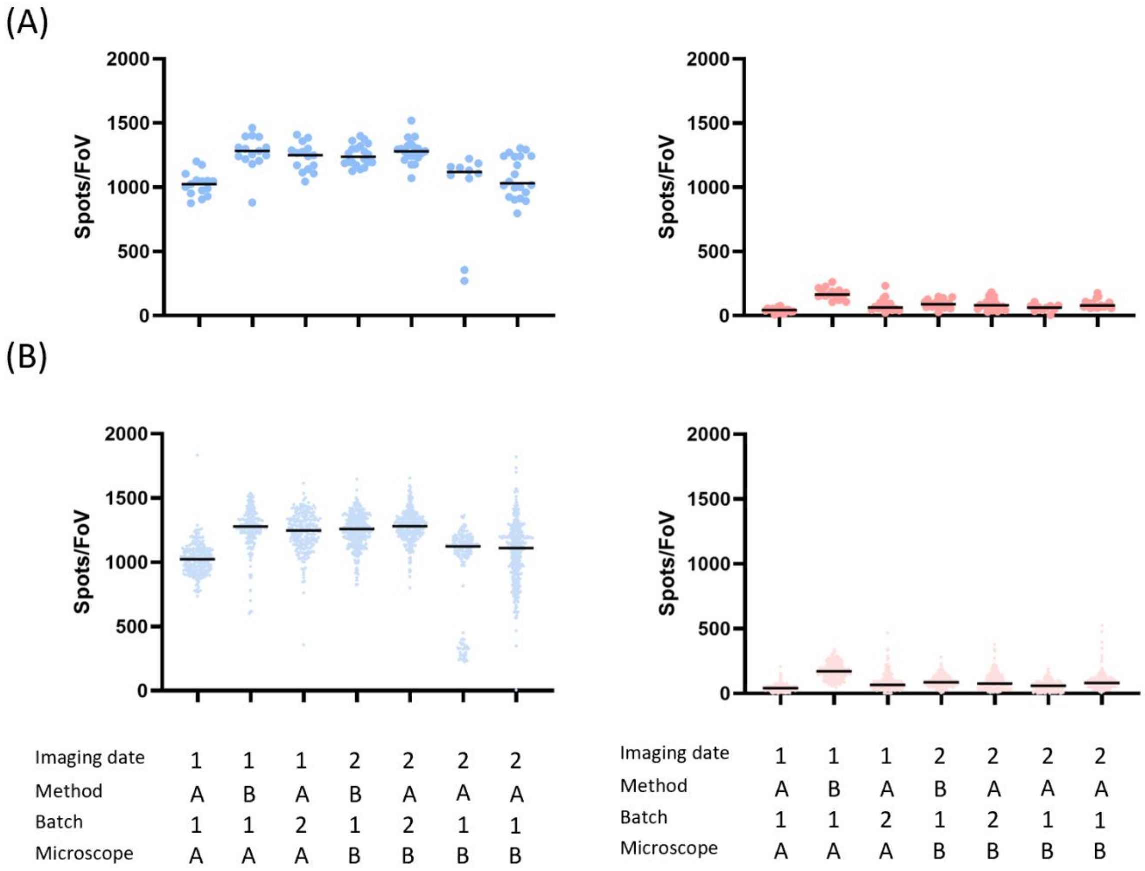

Figure S10 Reproducibility of surface capture and passivation. 7 coverslips were imaged on either different dates or with different instruments. The surface can be either cleaned by plasma (Method A) or organic solvent rinsing (Method B). The surface capture reproducibility was assessed by measuring the number of captured recombinant α-syn aggregates. The surface passivation reproducibility was assessed by measuring the number of non-specifically absorbed α-syn aggregates. The data points in panel (A) represent the averaged number detected in each imaging well. 16 FoV were collected for each well. The data points in panel (B) represent the number detected in each FoV. See the methods section for the differences between imaging set-up and coverslip preparation methods. 1840 images were taken in either the capture capability test or the passivation test. Each FoV contains an area of ~2500 μm^2.

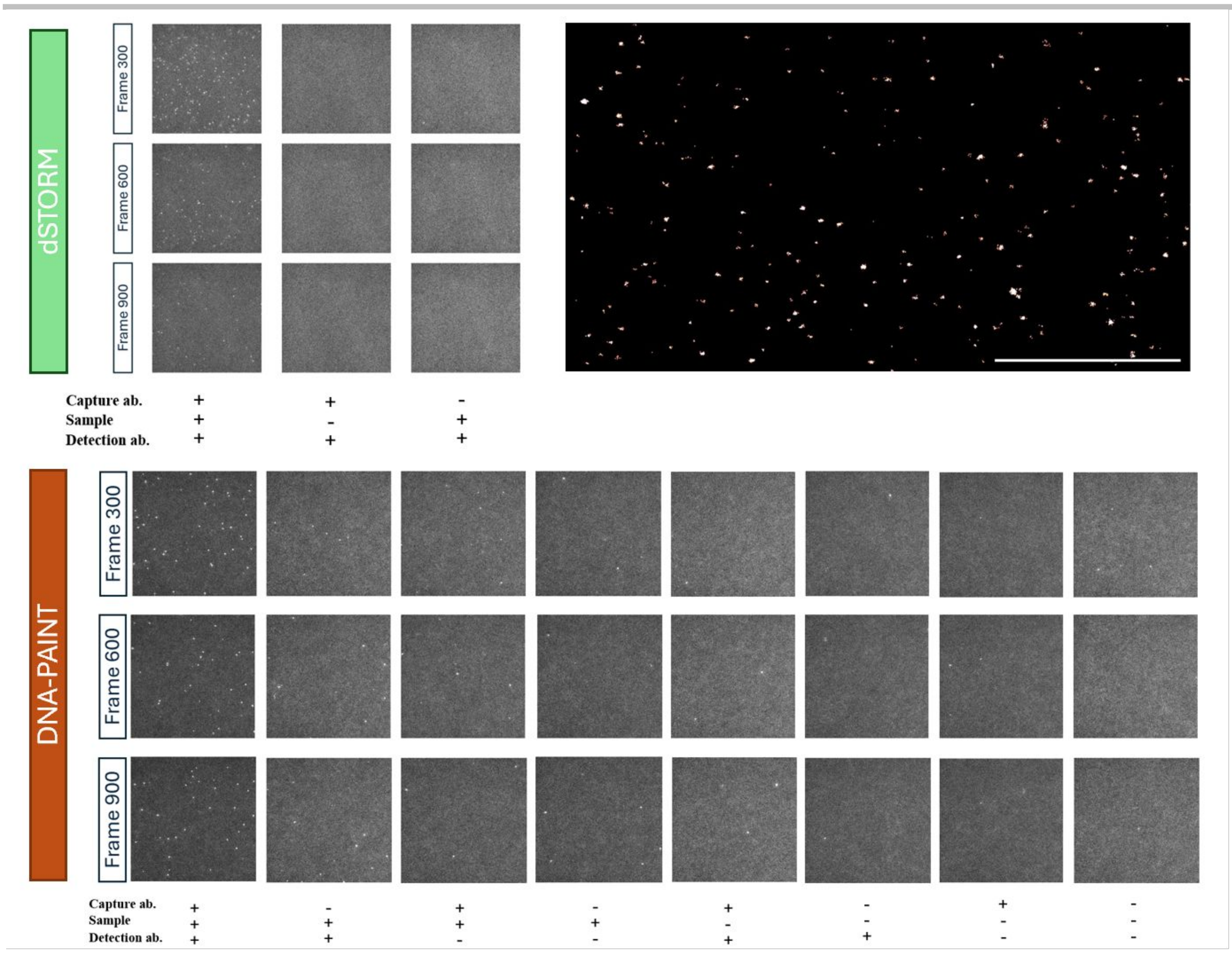

Figure S11 Representative single molecule images used for the super-resolution microscopy test. 3 Raw images taken at different time points were chosen for each condition tested. A super-resolved serum α-syn aggregates image (dSTORM with AF647 syn-211) was used as an example of the reconstructed image. Scale bar in super-resolution image: 5 µm.

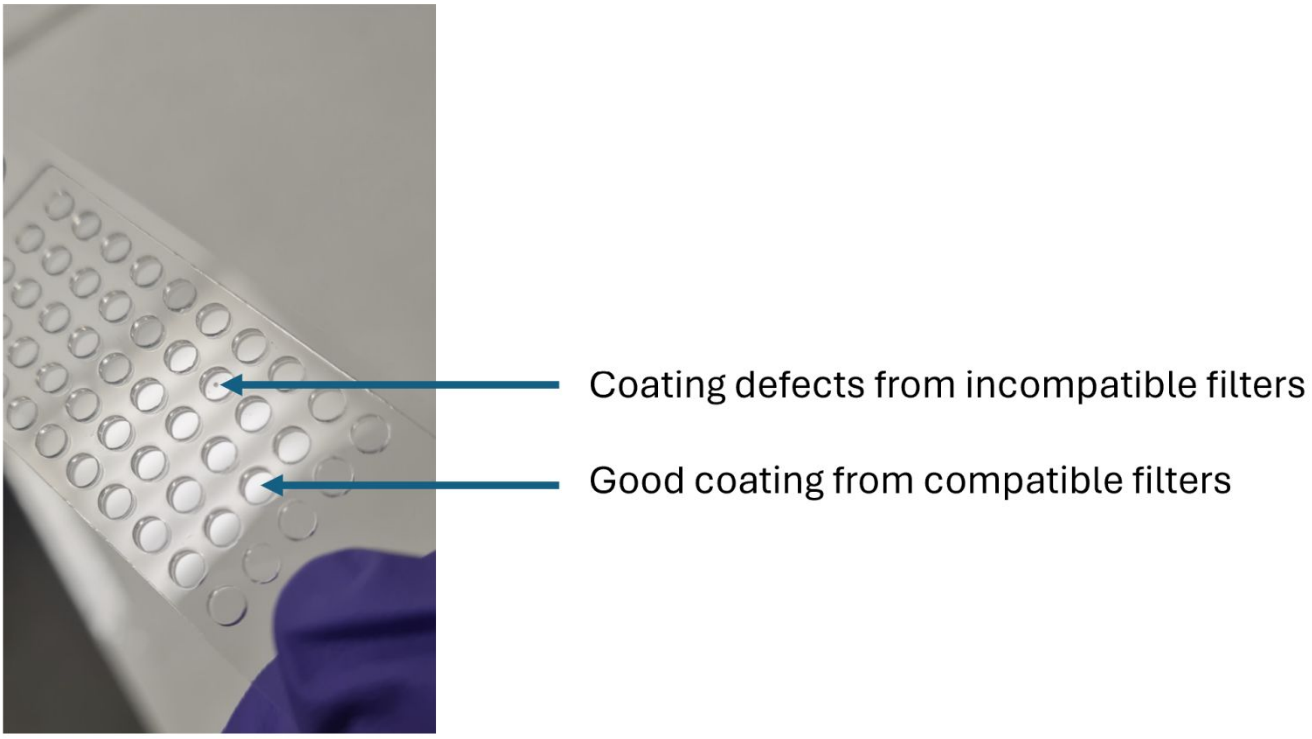

Figure S12 Representative photo of good and defective coatings. Coating defects (large residues) may appear on the surface if incompatible filters are used. This is likely due to the membrane compatibility with Rain-X.

Supplementary references
All supplementary references have also been cited in the main text

[1] W. Hoyer, T. Antony, D. Cherny, G. Heim, T.M. Jovin, V. Subramaniam. Dependence of α-synuclein aggregate morphology on solution conditions. *Journal of molecular biology*. **2002** 13;322(2):383-93.
[2] Y. P. Zhang, E. Lobanova, D. Emin, S.V. Lobanov, A. Kouli, C.H. Williams-Gray, & D. Klenerman *Analytical chemistry* **2023**, 95(41), 15254-15263.
[3] J. X. Meng, Y. Zhang, D. Saman, A. M. Haider, S. De, J. C. Sang, K. Brown, K. Jiang, J. Humphrey, L. Julian, E. Hidari, S. F. Lee, G. Balmus, R. A. Floto, C. E. Bryant, J. L. P. Benesch, Y. Ye, D. Klenerman, *Nat Commun* **2022**, *13*, 2692.
[4] L. Julian, J. C. Sang, Y. Wu, G. Meisl, J. H. Brelstaff, A. Miller, M. R. Cheetham, M. Vendruscolo, T. P. J. Knowles, F. S. Ruggeri, C. Bryant, S. Ros, K. M. Brindle, D. Klenerman, *Biophys J* **2022**, *121*, 4280–4298.
[5] M. Goedert, M. G. Spillantini, N. J. Cairns, R. A. Crowther, *Neuron* **1992**, *8*, 159–168.
[6] H. Gong, I. Holcomb, A. Ooi, X. Wang, D. Majonis, M. A. Unger, R. Ramakrishnan, *Bioconjug Chem* **2016**, *27*, 217–225.
[7] K. Kulenkampff, D. Emin, R. Staats, Y.P. Zhang, L. Sakhnini, A. Kouli, O. Rimon, E. Lobanova, C.H. Williams-Gray, F.A. Aprile, P. Sormanni, D. Klenerman, M. Vendruscolo. An antibody scanning method for the detection of α-synuclein oligomers in the serum of Parkinson's disease patients. Chemical Science. 2022;13(46):13815-28.
[8] K. Tsukakoshi, K. Abe, K. Sode, K. Ikebukuro. Selection of DNA aptamers that recognize α-synuclein oligomers using a competitive screening method. Analytical chemistry. 2012 Jul 3;84(13):5542-7.
